# Biosynthesis of Phosphatidylethanolamine and Phosphatidylcholine via the Kennedy Pathway in Cancer and Other Pathological Conditions

**DOI:** 10.3390/ijms27177519

**Published:** 2026-08-22

**Authors:** Jan Korbecki, Mateusz Bosiacki, Mikołaj Chlubek, Edyta Dzięciołowska-Baran, Michał Lubkowski, Krystyna Kabacińska, Katarzyna Barczak

**Affiliations:** 1 Institute of Health Sciences, Collegium Medicum, University of Zielona Gora, Zyty 28 St., 65-417 Zielona Gora, Poland; 2 Department of Biochemistry and Medical Chemistry, Pomeranian Medical University in Szczecin, Al. Powstańców Wlkp. 72, 70-111 Szczecin, Poland; mateusz.bosiacki@pum.edu.pl (M.B.); mikolaj.chlubek@pum.edu.pl (M.C.); 3 Department of Anatomy, Pomeranian Medical University in Szczecin, Al. Powstańców Wlkp. 72, 70-111 Szczecin, Poland; edyta.dzieciolowska.baran@pum.edu.pl; 4 Department of Functional Anatomy, Pomeranian Medical University in Szczecin, Al. Powstańców Wlkp. 72, 71-210 Szczecin, Poland; 5 Department of Endodontic Surgery, Pomeranian Medical University in Szczecin, Al. Powstańców Wlkp. 72, 70-111 Szczecin, Poland; krystyna.kabacinska@pum.edu.pl (K.K.); katarzyna.barczak@pum.edu.pl (K.B.)

**Keywords:** phospholipids, Kennedy pathway, phosphatidylcholine, phosphatidylethanolamine, cancer

## Abstract

The *de novo* biosynthesis of two major glycerophospholipids, phosphatidylethanolamine and phosphatidylcholine, occurs through the Kennedy pathway. In this process, ethanolamine or choline is initially phosphorylated to phosphoethanolamine or phosphocholine, respectively, and subsequently converted by cytidylyltransferases into CDP-ethanolamine and CDP-choline. Dysregulation of this pathway has been implicated in various diseases. This review provides a comprehensive overview of the enzymatic components of the Kennedy pathway and their physiological roles. Furthermore, it explores the involvement of these enzymes in diseases, with particular emphasis on cancer, while also considering other conditions such as type II diabetes, obesity, and liver disorders. To assess potential links between Kennedy pathway enzymes and cancer, a bioinformatics analysis was conducted using data from the GEPIA portal, covering 33 different cancer types.

## 1. Introduction

**Figure 1:**
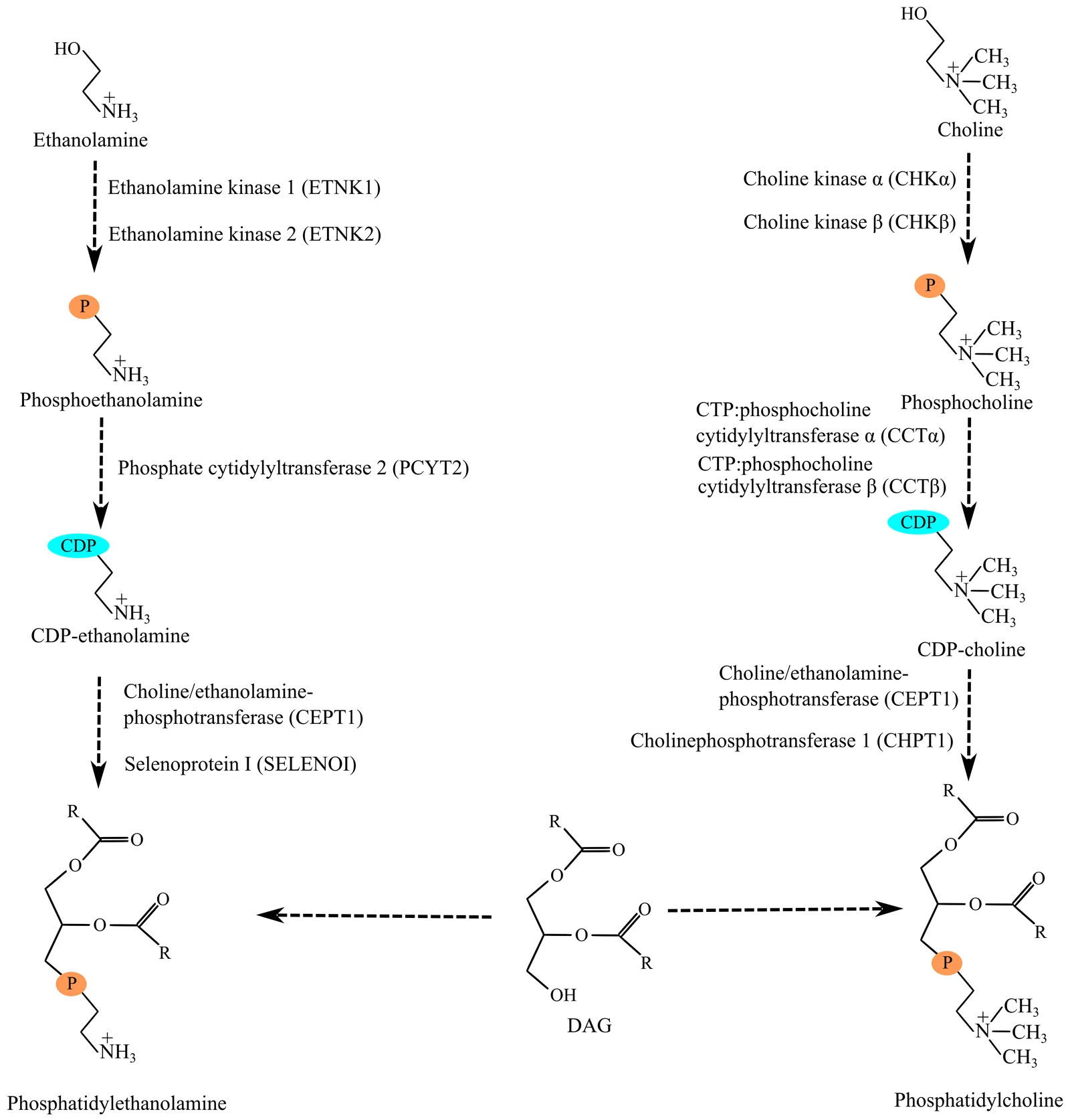
Biosynthesis of PE and PC via the Kennedy pathway. This pathway consists of three enzymatic steps. In the first step, choline or ethanolamine is phosphorylated by a specific kinase, resulting in the formation of phosphoethanolamine or phosphocholine. These intermediates are then converted by cytidylyltransferases into cytidine diphosphate (CDP)-ethanolamine and CDP-choline. In the final step, a specific transferase catalyzes the transfer of phosphoethanolamine or phosphocholine to diacylglycerol (DAG), leading to the synthesis of PE or PC.

Glycerophospholipids are fundamental structural components of all cells, forming the backbone of cellular membranes. They consist of a polyhydroxy alcohol backbone linked to fatty acids, a phosphate group, and, in some cases, additional molecules such as choline, serine, or ethanolamine [1]. Among them, phosphatidylethanolamine (PE) and phosphatidylcholine (PC) are synthesized *de novo* via the Kennedy pathway (Figure 1) [2]. This pathway comprises three sequential enzymatic reactions involving ten different enzymes. Dysregulation of this process has been linked to various diseases, including cancer, obesity, and diabetes.

**Figure 2:**
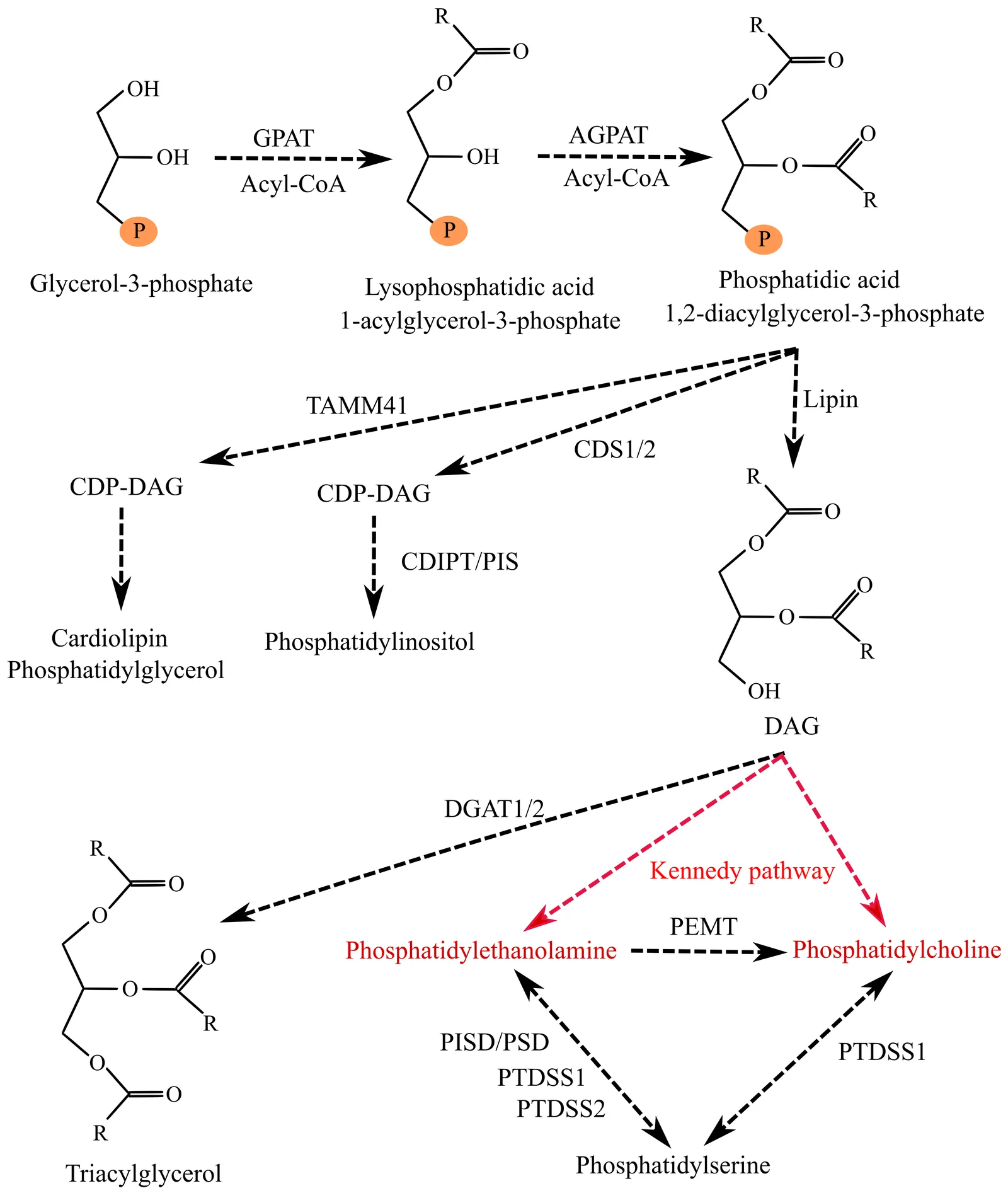
Interconnection of the Kennedy pathway with other biochemical pathways. The Kennedy pathway utilizes DAG as a substrate for the biosynthesis of PC and PE. However, DAG is also required for TAG synthesis. The relationship between the Kennedy pathway and overall cellular metabolism is even more complex because phosphatidic acid is also involved in the biosynthesis of other phospholipids. Phosphatidic acid is converted by lipins to DAG, which can subsequently enter the Kennedy pathway and be used for PE and PC biosynthesis. In addition, PE and PC can be interconverted through other metabolic reactions.

Like all biochemical processes, the Kennedy pathway is closely integrated with other metabolic pathways and should not be considered in isolation. It competes with diacylglycerol acyltransferase (DGAT) for DAG, thereby linking glycerophospholipid biosynthesis to triacylglycerol (TAG) synthesis [3]. Another important aspect is the interconversion of PC and PE through reactions involving phosphatidylethanolamine N-methyltransferase (PEMT), phosphatidylserine decarboxylase (PISD), and phosphatidylserine synthases (PTDSS1 and PTDSS2). These processes were discussed in detail in our previous review on the interconversion of phosphatidylserine (PS), PE, and PC [4]. Thus, the biosynthesis of TAG, PE, and PC forms a closely interconnected metabolic network (Figure 2). Accordingly, the Kennedy pathway discussed in this review should be viewed within the broader context of cellular lipid metabolism.

This review aims to examine the relationship between the enzymes of the Kennedy pathway and human diseases. A deeper understanding of these associations may help identify potential therapeutic targets for various disorders.

## 2. Biosynthesis of Phosphatidylethanolamine via the Kennedy Pathway

### 2.1. Ethanolamine Kinases

The synthesis of PE or PC occurs through the Kennedy pathway [2], comprising three distinct reactions. In the first step, during the synthesis of PE, phosphorylation of ethanolamine takes place, resulting in the formation of phosphoethanolamine. In humans, two enzymes with ethanolamine kinase (ETNK) activity, ETNK1 [5] and ETNK2 [6], are responsible for this reaction. ETNK1 does not show choline kinase activity [5] and its expression occurs at similar levels in all organs [7]. On the other hand, ETNK2 has ethanolamine kinase activity and may also have slight choline kinase activity [6]. The highest expression of this enzyme is found in the testis, liver, and kidney [7]. The pathway for synthesizing PE within the Kennedy pathway plays a crucial role in the differentiation of T follicular helper (TFH) cells, as highlighted by Fu et al. in 2021 [8]. Particularly, PE is indispensable for the differentiation of TFH cells and the presence of CXC motif chemokine receptor 5 (CXCR5) on their cell membrane. TFH cells play a pivotal role in the functioning of B cells. Consequently, the *de novo* synthesis of PE is a vital factor in shaping humoral immunity.

#### Ethanolamine Kinases in Cancers

The *ETNK1* gene mutations identified in tumors contribute to an increased incidence of subsequent mutations in cancer cells, as elucidated by Fontana et al. in 2020 [9]. This phenomenon is linked to heightened mitochondrial activity, where phosphoethanolamine acts as an inhibitor of succinate dehydrogenase (SDH), also known as mitochondrial complex II. Mutations in *ETNK1* diminish phosphoethanolamine production, consequently elevating mitochondrial activity and reactive oxygen species (ROS) production. The ensuing ROS inflict damage to DNA, perpetuating a cycle of mutagenesis [9].

ETNKs emerge as pivotal players in various cancer types. Studies on gastric cancer underscore the downregulation of ETNK1 expression by miR-708-3p [10] and miR-199a-3p [11], which is crucial for the proliferation and migration of gastric cancer cells, as well as tumor growth. Significantly, the opposing role of the second isoform, ETNK2, is evident in gastric cancer, where its increased expression correlates with advanced stages and poorer prognoses [12]. Moreover, higher ETNK2 expression may contribute to hepatic metastasis in gastric cancer [12]. The two enzymes likely play distinct roles in different processes in gastric cancer and also exhibit different expression patterns. This may account for the opposing findings reported for the two isoforms in this type of cancer.

In renal clear cell carcinoma, the downregulation of ETNK1 expression may be modulated by LncRNA FEZf1-as1 [13]. Consequently, the reduced expression of ETNK1 in renal clear cell carcinoma, as observed by Lou et al. in 2023 [13], contradicts the data on the Gene Expression Profiling Interactive Analysis (GEPIA) portal [14]. Meanwhile, the expression of the second isoform, ETNK2, is also diminished in renal clear cell carcinoma [14,15]. Lower expression of both ETNKs in renal clear cell carcinoma correlates with unfavorable patient outcomes, indicative of their potential anti-tumor role [14,15].

Lung adenocarcinoma exhibits an intriguing profile with increased *de novo* synthesis of PE but reduced PC synthesis compared with healthy lung tissue. This is substantiated by the distinct expression patterns of enzymes involved in the *de novo* phospholipid synthesis pathways [14,16]. Specifically, heightened expressions of ETNK2 and selenoprotein I (SELENOI—another name for ethanolaminephosphotransferase 1 (EPT1)) are observed [16]. SELENOI exhibits CDP-ethanolamine:1,2-diacylglycerol ethanolaminephosphotransferase activity and catalyzes the third step of the Kennedy pathway for PE biosynthesis. Thus, in lung adenocarcinoma tumors, PE production via the Kennedy pathway appears to be enhanced at two of the three steps of this pathway.

Elevated ETNK1 or SELENOI expression in tumors is linked to poorer prognoses for lung adenocarcinoma patients [14]. Conversely, higher expression of choline kinase α (*CHKA*), a gene encoding the enzyme responsible for the initial step in PC biosynthesis, correlates with improved patient outcomes [14]. This suggests a pivotal role for the PE synthesis pathway in lung adenocarcinoma tumorigenesis, while the PC synthesis pathway may exert anti-tumor effects.

Papillary thyroid carcinoma highlights the involvement of ETNK2 in tumorigenic processes [17]. Increased ETNK2 expression in papillary thyroid carcinoma tumors is associated with poorer prognoses. This elevated expression is linked to the stimulation of proliferation and migration of cancer cells through activation of Hippo and epithelial–mesenchymal transition (EMT) pathways [17].

ETNKs are also implicated in hematolymphoid tumors, particularly myeloid neoplasms, such as myelodysplastic syndrome, myelodysplastic/myeloproliferative neoplasm, and acute myeloid leukemia (AML), where *ETNK1* gene mutations are prevalent [18]. Around 16% of atypical chronic myeloid leukemia (aCML) cases exhibit mutations in the *ETNK1* gene [19]. These mutations result in reduced PE production, suggesting their significant role in aCML tumorigenesis. *Ex vivo* experiments on aCML cells have shown that phosphoethanolamine reduces the growth of colonies of these cells, pointing towards potential therapeutic applications [9].

The PE synthesis pathway emerges as a crucial factor in skin cancers, particularly in basal cell carcinomas [20]. Notably, Gorlin syndrome mutations in the Patched 1 (*PTCH1*) gene lead to the formation of basal cell carcinomas. The resistance observed in targeted drugs within the hedgehog pathway is linked to basal-to-squamous cell carcinoma transition (BST). This transition can be induced by mutations in the PE synthesis pathway genes, such as PCYT2 or ETNK1 [20].

These studies collectively underscore the intricate involvement of ETNKs in tumorigenesis. Depending on the specific tumor type, a given ETNK may exhibit pro- [17] or anti-tumor effects [19]. Tumors with *ETNK* mutations or decreased expression, where decreased enzyme activity correlates with poorer prognoses, may benefit from therapeutic interventions involving intermediates in the Kennedy pathway, such as phosphoethanolamine. The *ex vivo* studies on aCML suggest promising therapeutic potential in this regard [19]. In cases where ETNKs are implicated in tumorigenesis, and higher expression correlates with worse prognoses, the development of ETNK inhibitors becomes a potential avenue for therapeutic exploration. Presently, these inhibitors are not known and warrant further investigation and development.

### 2.2. Phosphoethanolamine Cytidylyltransferase

Phosphoethanolamine is converted to CDP-ethanolamine by phosphate cytidylyltransferase (PCYT)2, also known as cytidine triphosphate (CTP):phosphoethanolamine cytidylyltransferase (ECT) [2,21,22]. This enzyme demonstrates a high degree of specificity for phosphoethanolamine and exhibits modest activity towards phosphocholine [23].

PCYT2 is predominantly localized in the cytoplasm [24], although some sources also report its localization in the endoplasmic reticulum [25]. PCYT2 has not been detected in the nucleus, mitochondria, or peroxisomes [25]. Through alternative splicing, PCYT2 generates diverse isoforms [26,27,28], each characterized by distinct catalytic parameters and regulatory features. Notably, in mice, PCYT2β displays twofold higher activity than PCYT2α [26]. Moreover, the identification of PCYT2γ in mice reveals its interaction with other PCYT2 isoforms, resulting in reduced overall activity [28]. The isoforms may exist as homo- or heterodimers, suggesting a potential mechanism for regulating their activity [27]. Further modulation occurs through phosphorylation, where protein kinase C (PKC)α, PKCβI, and PKCβII phosphorylate PCYT2α at Ser^215^ and Ser^223^, augmenting its activity [28].

The biosynthesis of PE assumes paramount importance in muscular physiology. Myoblast differentiation prompts an increase in the expression of the aforementioned enzyme, thereby facilitating muscular development [29]. Conversely, the aging process induces a decline in PCYT2 activity within muscles, culminating in muscle weakness [30]. Beyond its role in muscle biology, the metabolic fate of phosphoethanolamine holds pivotal significance in the development and sustenance of the nervous system. Mutations in the *PCYT2* gene instigate disruptions in etherphospholipid metabolism, contributing to congenital neurological disorders categorized as complex hereditary spastic paraplegia or axonal polyneuropathy [31,32].

#### 2.2.1. Phosphoethanolamine Cytidylyltransferase in Cancers

PCYT2 may exert a crucial role in oncogenic processes. When faced with nutrient deficiencies, particularly in glutamine, the expression of this enzyme diminishes in cancer cells [33]. This becomes particularly significant due to tumor regions experiencing chronic hypoxia, situated far from blood vessels, resulting in the accumulation of phosphoethanolamine in cancer cells. This compound facilitates the growth of cancer cells under conditions of nutrient scarcity. PCYT2 also has the capacity to biosynthesize CDP-glycerol from glycerol-3-phosphate and CTP [34]. CDP-glycerol, in turn, reduces the glycosylation of α-dystroglycan [35], a cell-surface protein, ultimately weakening cell adhesion [36]. This phenomenon is observed in tumors, particularly in colorectal cancer, contributing to cancer cell migration and metastasis.

**Figure 3:**
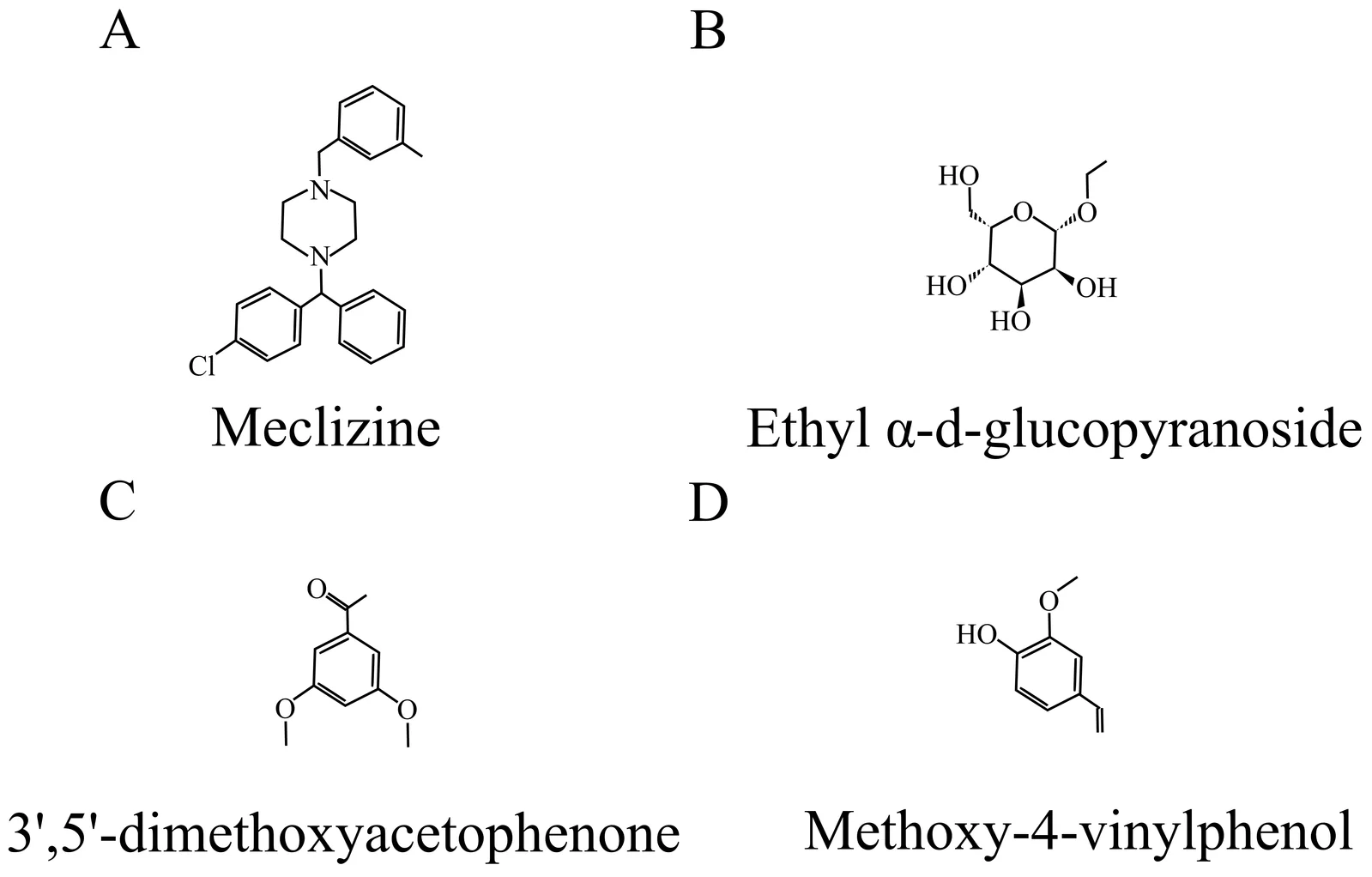
PCYT inhibitors. (**A**) Meclizine and compounds isolated from *Citrus sinensis* peel: (**B**) ethyl α-d-glucopyranoside, (**C**) 3′,5′-dimethoxyacetophenone, and (**D**) methoxy-4-vinylphenol.

Meclizine has been shown to be a potential PCYT2 inhibitor (Figure 3) [37]. Widely used to treat motion sickness due to its antihistamine properties, meclizine is currently being explored as a potential anticancer drug [38]. Particularly when combined with PFK158, an inhibitor of 6-phosphofructo-2-kinase/fructose-2,6-bisphosphatase 3 (PFKFB3), *in vitro* and *in vivo* experiments have demonstrated efficacy against hepatocellular carcinomas and *in vitro* in acute myeloid leukemia [39]. Additionally, recent findings reveal another PCYT inhibitor present in Citrus sinensis peel, although its potential anticancer properties remain unexplored [40].

#### 2.2.2. Phosphoethanolamine Cytidylyltransferase in Other Diseases

PCYT2 is also implicated in type II diabetes. Research conducted on mice revealed a decrease in PCYT2 expression in the liver during diabetes [41]. Consequently, this leads to mitochondrial dysfunction and liver cell damage. Notably, CDP-ethanolamine emerges as a potential therapeutic compound mitigating the impact of diabetes on the liver.

Studies involving mice with induced defects in the *PCYT2* gene underscore the significance of phosphoethanolamine metabolism in overall metabolic function. This observation was observed in mice with only one functional copy of the *PCYT2* gene [42]. The absence of functional *PCYT2* alleles proved embryonically lethal. Diminished PCYT2 activity led to accumulation of substrates catalyzed by this enzyme, resulting in an accumulation of phosphoethanolamine—an inhibitor of mitochondrial respiration [37].

Reduced PCYT2 activity also contributed to increased utilization of DAG for TAG production and an elevated *de novo* fatty acid production [43]. These metabolic alterations contributed to obesity and insulin resistance, as shown in studies of obese individuals [44]. Adipose tissue exhibited a negative correlation between PCYT2 expression and Body Mass Index (BMI) and waist-to-hip ratio, implying that reduced PCYT2 activity may contribute to obesity [45]. Animal studies further demonstrated that diminished PCYT2 activity in males led to hypertension and cardiac hypertrophy. In older mice, altered metabolism resulted in nonalcoholic steatohepatitis [46].

The ramifications of reduced PCYT2 activity offer insights into anticipating potential side effects of drugs targeting this enzyme. Furthermore, they may illuminate the genetic underpinnings of certain diseases, such as type II diabetes, obesity, cardiovascular diseases, and nonalcoholic steatohepatitis, where diminished enzyme activity could heighten susceptibility to these conditions.

### 2.3. Ethanolaminephosphotransferases

In the third reaction of the Kennedy pathway, PE is synthesized from CDP-ethanolamine and DAG [2]. The enzyme catalyzing this reaction possesses CDP-ethanolamine:1,2-diacylglycerol ethanolaminephosphotransferase activity. These enzymes are choline/ethanolaminephosphotransferases (CEPT1) [47,48] and SELENOI [49]. It should be noted that CEPT1 also exhibits cholinephosphotransferase activity, which is more prominent than in the synthesis of PE, thus enabling its involvement in PC synthesis as well [47]. CEPT1 exhibits the highest activity toward 1-palmitoyl-2-oleyl-glycerol when the substrate is CDP-ethanolamine [48]. CEPT1 is a membrane protein located in the endoplasmic reticulum and nuclear membranes [50,51]. On the other hand, SELENOI is an ethanolaminephosphotransferase enzyme that produces PE [49].

SELENOI and CEPT1 differ in terms of the type of PE produced. SELENOI is responsible for synthesizing PE with longer fatty acid chains, such as PE 36:1 and PE 40:5 [51]. CEPT1, on the other hand, is responsible for synthesizing PE with shorter fatty acid chains, such as PE 32:1 and PE 34:2 [51]. SELENOI also shows greater activity toward 1-alkyl-2-acylglycerol [52,53], which leads to the production of plasmanyl-PE. Therefore, this enzyme is involved in the synthesis of ether glycerophospholipids, such as plasmalogens.

SELENOI is more important than CEPT1 in the synthesis of plasmanyl-PE [51]. In contrast to CEPT1, SELENOI does not exhibit cholinephosphotransferase activity [49]. In the cell, SELENOI is located in the Golgi apparatus [51], in a different location than CEPT1. The highest expression of SELENOI is found in the brain, particularly in the cerebellum, as well as in the liver and pancreas. In the brain, SELENOI plays an important role in lipid synthesis [52]. Mutations in the gene encoding this enzyme lead to brain neurodegeneration, including hereditary spastic paraplegia [53,54]. This protein is also important in T-cell differentiation [55,56].

#### 2.3.1. Ethanolaminephosphotransferases in Cancers

To date, the role of ethanolaminephosphotransferases in cancer remains insufficiently explored. In high-grade serous ovarian cancer, CEPT1 appears to be linked to heightened tumor aggressiveness, with evidence indicating that increased gene expression correlates with tumor recurrence [57]. However, the precise relationship between CEPT1 and treatment resistance in high-grade serous ovarian cancer remains elusive.

The expression of CEPT1 shows an increase in non-Hodgkin lymphoma (NHL) tumors compared to healthy lymph node tissue [58]. Nevertheless, the contribution of this enzyme to the oncogenic processes of NHL has yet to be thoroughly investigated.

Additionally, a study highlights a higher expression level of SELENOI in melanoma cells compared to normal melanocyte cells [59]. However, again, the exact role of this enzyme in the oncogenic mechanisms of melanoma remains unknown.

As previously discussed, lung adenocarcinoma exhibits heightened expression of enzymes in the Kennedy pathway involved in PE biosynthesis, specifically ETNK2 and SELENOI, in comparison to healthy lung tissue [16]. The elevated expression of these genes is correlated with a less favorable prognosis for patients with lung adenocarcinoma [14]. This implies that the PE synthesis pathway in lung adenocarcinoma significantly influences oncogenic processes.

#### 2.3.2. Ethanolaminephosphotransferases in Other Diseases

Research on mice has demonstrated that CEPT1 expression in muscles increases during obesity [60]. Elevated expression of this gene results in muscle insulin resistance, while reduced CEPT1 expression enhances insulin responsiveness in skeletal muscles. These findings have been corroborated in human studies [60]. There exists a negative correlation between the level of CEPT1 expression in muscles and insulin sensitivity. Consequently, CEPT1 emerges as an intriguing therapeutic target for addressing obesity and insulin resistance associated with obesity.

Variants of the CEPT1 gene have been identified as risk factors for male-pattern hair loss (MPHL) [61]. However, the details of this association remain unclear.

## 3. Biosynthesis of Phosphatidylcholine via the Kennedy Pathway

### 3.1. Choline Kinase

We recently published a review on choline kinase, which provides a detailed exploration of this enzyme [62]. Readers seeking an in-depth understanding are encouraged to consult that article. In this paper, we present a concise overview of choline kinase, summarizing its function and its relevance to various diseases.

The first step in PC biosynthesis involves the phosphorylation of choline to phosphocholine, a reaction catalyzed by choline kinase α (CHKα) and choline kinase β (CHKβ) [63]. These two enzymes play distinct roles in metabolism: CHKβ is primarily involved in PE synthesis, whereas CHKα contributes to both PC and PE biosynthesis [64]. Additionally, alternative splicing of the CHKA gene gives rise to two isoforms, CHKα1 and CHKα2 [65]. In mice, CHKα2 is 18 amino acids longer than CHKα1 [63]. While both isoforms exhibit similar tissue expression patterns, their functional differences remain unclear [65].

The activity and expression of choline kinases are tightly regulated, particularly through phosphorylation, which enhances their enzymatic activity. CHKα2, for instance, is phosphorylated by c-Src, while CHKβ is regulated by protein kinase A (PKA) [66,67].

Both CHKα and CHKβ fulfill essential physiological functions, though their roles vary across tissues. CHKα is particularly important in neural tissue, and mutations in the CHKA gene are associated with neurodevelopmental disorders, including epilepsy and microcephaly [68,69,70]. In contrast, CHKβ plays a crucial role in bone and muscle function [71,72,73]. Mutations in the CHKB gene result in megaconial congenital muscular dystrophy [72,73].

#### Choline Kinase in Cancer

CHKα is widely recognized as an oncogene in various cancers, whereas CHKβ is considered a tumor suppressor. Elevated CHKα expression in tumors is correlated with poor prognosis in several malignancies, including esophageal squamous cell carcinoma [74], hepatocellular carcinoma [75], non-small-cell lung cancer [76], and prostate cancer [77].

The oncogenic role of CHKα is linked to multiple mechanisms. As a key enzyme in PC biosynthesis, CHKα provides essential structural components for rapidly proliferating cells, including cancer cells. This may explain why Ras proteins upregulate choline kinase activity [78,79]. Additionally, CHKα localizes to the nucleus, where it participates in PC production, influencing chromatin organization and gene expression [80,81].

Beyond its role in phospholipid metabolism, CHKα contributes to cancer progression through kinase-independent mechanisms. It enhances EGFR signaling by promoting its interaction with mechanistic target of rapamycin complex (mTORC)2 [66,82] and can directly phosphorylate protein kinase B (PKB)/Akt [83], leading to Akt activation and increased cell proliferation. Moreover, CHKα functions as a chaperone for the androgen receptor (AR), stabilizing it and promoting its expression [84]. This is particularly significant in prostate cancer, where AR plays a key role in tumor growth.

A broad range of CHKα inhibitors has been identified, with several extensively studied compounds, including EB-3D [85], MN58b [86], ICL-CCIC-0019 [87], RSM932A [86], and PL48 [88]. However, these represent only a fraction of the known CHKα inhibitors, as numerous additional candidates have been investigated. Many of these inhibitors exhibit potent *in vitro* activity at submicromolar concentrations, demonstrating anticancer effects, though they have not yet advanced to clinical trials.

**Figure 4:**
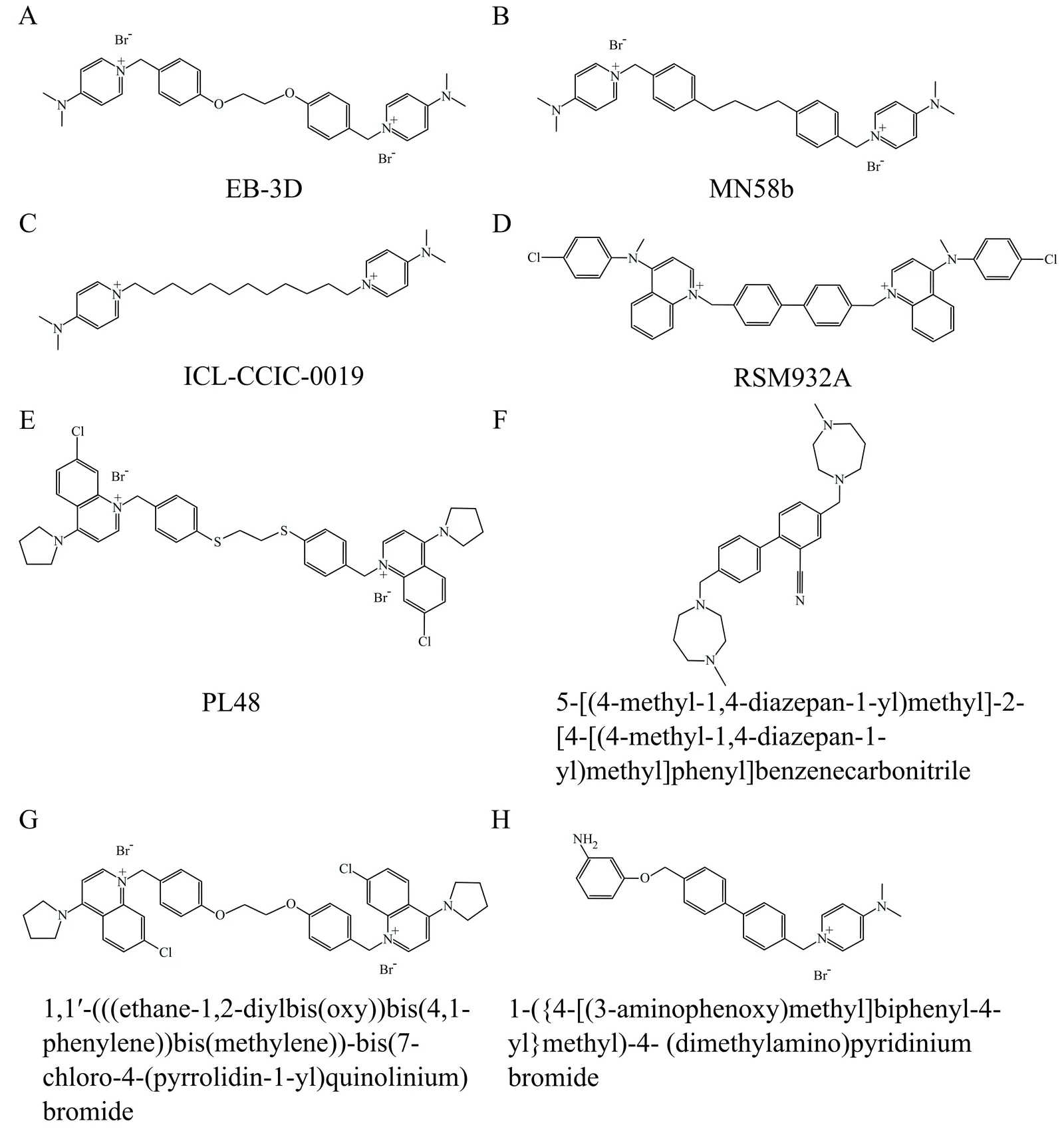
Selected CHKα inhibitors: (**A**) EB-3D, (**B**) MN58b, (**C**) ICL-CCIC-0019, (**D**) RSM932A, (**E**) PL48, (**F**) 5-[(4-methyl-1,4-diazepan-1-yl)methyl]-2-[4-[(4-methyl-1,4-diazepan-1-yl)methyl]phenyl]benzenecarbonitrile, (**G**) 1,1′-(((ethane-1,2-diylbis(oxy))bis(4,1-phenylene))bis(methylene))-bis(7-chloro-4-(pyrrolidin-1-yl)quinolinium) bromide, (**H**) 1-({4-[(3-aminophenoxy)methyl]biphenyl-4-yl}methyl)-4-(dimethylamino)pyridinium bromide.

Examples of promising CHKα inhibitors include the following:

• 5-[(4-methyl-1,4-diazepan-1-yl)methyl]-2-[4-[(4-methyl-1,4-diazepan-1-yl)methyl]phenyl]benzenecarbonitrile [89];
• 1,1′-(((ethane-1,2-diylbis(oxy))bis(4,1-phenylene))bis(methylene))-bis(7-chloro-4-(pyrrolidin-1-yl)quinolinium) bromide [90];
• 1-({4-[(3-aminophenoxy)methyl]biphenyl-4-yl}methyl)-4-(dimethylamino)pyridinium bromide [91] (Figure 4).

However, the inhibitors described above have not yet undergone clinical evaluation as anticancer agents. One important limitation is that CHKα plays an essential role in normal cellular and physiological functions, and its inhibition may therefore result in adverse effects [62]. Moreover, analysis using the GEPIA database shows that higher CHKα expression is associated with poorer prognosis in only 3 of 33 cancer types [14]. These findings suggest that the therapeutic potential of CHKα inhibitors may be limited to selected malignancies rather than extending across a broad spectrum of cancers.

### 3.2. Phosphocholine Cytidylyltransferases

In the second step of the Kennedy pathway, analogous to PE synthesis, phosphocholine undergoes conversion to CDP-choline catalyzed by CTP:phosphocholine cytidylyltransferase (CCT)α/PCYT1A [92] and CCTβ/PCYT1B [93]. Studies on CCTα reveal autoinhibition, wherein both enzymes possess a lipid-binding domain and a phosphorylation region inhibiting their activity [94,95,96]. These enzymes sense membrane curvature strain in the endoplasmic reticulum induced by elevated levels of DAG or unsaturated PE [97]. Dephosphorylation of the enzymes enhances their binding to lipid membranes in the cell [98,99]. Subsequently, CCTα and CCTβ associate with lipid membranes in the cell, allowing for PC biosynthesis, leading to an elevation in the PC levels within cell membranes and the maintenance of homeostasis.

Factors influencing the activity of CCTα include sphingosine, which reduces CCTα expression [100]. Ceramides also exhibit this effect as they are transformed into sphingosine, consequently diminishing CCT activity [101]. This is dependent on cytosolic phospholipase A_2_ (cPLA_2_) activation and lysophosphatidylcholine (lysoPC) generation. Another factor enhancing CCT activity is glucosylceramide [102]. Pathological accumulation of glucosylceramide in cells, including neuronal cells, occurs in Gaucher disease. The mentioned effect of glucosylceramide may play a role in pathological mechanisms of this disease [102].

CCTα is localized in both the endoplasmic reticulum and the nucleus, specifically the nuclear envelope and nuclear lipid droplets [99,103]. The nuclear localization signal (NLS) and monoubiquitination at Lys^57^, which is in close proximity to the NLS, are responsible for CCTα localization [104]. Monoubiquitination of CCTα leads to its export from the cell nucleus to the cytoplasm and proteolytic degradation of the enzyme [105]. Calpain can degrade CCTα, but calmodulin binding to CCTα protects it from calpain-mediated degradation [105]. Tumor necrosis factor-α (TNF-α) increases CCTα protein degradation, which is relevant in inflammatory reactions [106].

The intracellular localization of CCTα plays a crucial role in its functions. In quiescent cells, CCTα is located in the cell nucleus [107], while in dividing cells, especially during exit from the G_0_ phase and in the G_1_ phase, it is found in the cytoplasm [107,108]. However, another study indicates that CCTα is located in the cell nucleus in S phase and in the cytoplasm in G_0_ phase [109]. In the nucleus, CCTα participates in the Kennedy pathway biosynthesis of PC, which is crucial for chromatin structure associated with PC and for the functioning of the nucleoplasmic reticulum [110,111]. CCTα expression increases in the S phase due to specificity protein 1 (Sp1) [109,112], phosphorylated by cyclin-dependent kinase 2 (CDK2) [109]. This phosphorylated Sp1 binds to the promoter of the *PCYT1A* gene, enhancing CCTα expression. The highest CCT activity occurs in the G_1_ phase of the cell cycle, ensuring elevated CCTα expression in the S phase and preparing cellular metabolism for structural component synthesis post-mitosis [108]. Caspase 3-mediated removal of the N-terminal portion containing NLS during apoptosis increases CCTα activity [113], but this process reduces PC synthesis in the Kennedy pathway by limiting choline uptake [114].

CCTα expression is observed in all organs, and alterations in its expression or activities lead to changes in various organs [115]. CCTα serves multiple physiological functions, including regulating lipid droplet size, participating in autophagy [116], and contributing to PC production in the liver, influencing blood PC, high-density lipoprotein (HDL), and very low-density lipoprotein (VLDL) levels [117]. Decreased liver CCTα activity leads to TAG accumulation. CCTα is crucial for B cell function [118] and plays a role in lipid absorption in the intestinal epithelium during a high-fat diet [119]. Reduced CCTα expression in the intestines results in barrier function disturbances and loss of goblet cells, potentially relevant in obesity treatment [120].

CCTα is significant in human development. The *PCYT1A* SNP rs939883 genotype AA in women is associated with increased spina bifida risk [121]. Mutations in the *PCYT1A* gene lead to congenital lipodystrophy, severe fatty liver disease with diabetes [122], spondylometaphyseal dysplasia with cone-rod dystrophy [123,124], and isolated retinal dystrophy [125]. The E280del mutation in CCTα is linked to increased activity and leads to congenital lipodystrophy and severe fatty liver disease with diabetes [122]. Other mutations reducing CCTα activity are also associated with this disease. Mutations in other genes encoding enzymes in the PC synthesis pathway lead to different genetic disorders.

The gene *PCYT1B* produces three CCTβ protein variants via alternative splicing: CCTβ1, CCTβ2 [103], and CCTβ3 [115]. CCTβ is located in the endoplasmic reticulum [103]. CCTβ3 is essential for autophagy induced by cellular starvation [126]. This enzyme synthesizes PC, contributing to autophagosome membrane formation. CCTβ3 is crucial for starvation-induced lipid droplet function, enabling further autophagy by utilizing lipid droplet components for PC synthesis.

CCTβ exhibits the highest expression in the brain, with lower expression in the testis [115]. Organ-specific localization of these enzymes is significant for the consequences of changes in their expression or disruptions in enzyme activity. Studies on mice show that CCTβ2 is essential for proper ovarian and testicular function, with decreased expression leading to reduced fertility. CCTβ2 is also necessary for the proper functioning of the nervous system, contributing to PC synthesis in distal axons [127] and participating in neurite outgrowth and branching [128].

#### 3.2.1. Phosphocholine Cytidylyltransferases in Cancers

CCT assumes a noteworthy role in various neoplastic conditions. CCTα’s influence on genome stability is exemplified by its involvement in Bcl-2-mediated tetraploidization of cells [129]. This phenomenon, independent of the catalytic attributes of CCTα, is particularly pertinent in the context of tumorigenesis. Tetraploid cells serve as a transitional state between normal and cancer cells [130,131]. Throughout tumorigenesis, normal cells undergo distinct pathological events, such as cell fusion or mitotic aberrations, leading to the transformation into tetraploid cells. This precarious state culminates in chromosomal alterations, ultimately facilitating the conversion of these cells into cancerous entities.

In diverse tumor types, heightened expression of CCTα and CCTβ is discerned, orchestrated by various factors. Notably, Ha-Ras-induced elevation of CCTα and CCTβ2 in cancer cells is contingent upon the activation of extracellular signal-regulated kinase (ERK), mitogen-activated protein kinase (MAPK), and specificity protein 3 (Sp3) [113,132]. Concurrently, Ha-Ras exerts regulatory effects on CCT activity, suppressing it while fostering the activity of choline kinase and choline phosphotransferase [132,133]. Consequently, this dual influence results in either diminished [133] or unaltered [113] PC levels in comparison to cells devoid of H-ras overexpression. Furthermore, in detached cells, CCTα activation enhances PC synthesis, thereby promoting the proliferation and anchorage-independent growth of ras-transformed cells [113].

The regulatory impact on CCTα expression extends to RNA methylation processes, as evidenced by the upregulation induced by 5-methylcytosine (m5C) RNA methyltransferase NSUN2, particularly in gastric cancer cells [134].

The significance of CCTα and CCTβ in cancer has undergone only cursory examination, and the precise role of each enzyme in tumorigenesis remains nuanced. CCTα’s impact can sway between an anti-oncogenic and pro-oncogenic influence, contingent upon the specific cancer type. Enhanced CCTα expression in bladder cancer post-cystectomy [135] and in lung adenocarcinoma [136] aligns with more favorable prognoses, while its heightened presence in gastric cancer correlates with adverse outcomes [134]. Bioinformatic analysis on the GEPIA platform suggests a potential pro-oncogenic character of CCTα, as elevated expression associates with worse prognoses in five cancer types (breast invasive carcinoma, kidney chromophobe, liver hepatocellular carcinoma, pancreatic adenocarcinoma, prostate adenocarcinoma) and better prognoses in one cancer type (kidney renal clear cell carcinoma) [14]. Conversely, increased CCTβ expression in hepatocellular carcinoma is linked with improved prognoses [137], with GEPIA indicating worse prognoses in four cancer types (bladder urothelial carcinoma, colon adenocarcinoma, stomach adenocarcinoma, thyroid carcinoma) and better prognoses in two types (adrenocortical carcinoma, sarcoma), emphasizing a prevailing pro-oncogenic nature of CCTβ [14].

The exact involvement of CCTα and CCTβ in tumorigenesis has received focused attention in specific cancer types. In lung adenocarcinoma, CCTα demonstrates anti-tumor responses, impeding the proliferation and migration of cancer cells [136], substantiated by a reduction in mTORC1 activation [107]. Consequently, heightened CCTα expression in lung adenocarcinoma correlates with improved prognoses [136], mirroring observations for other Kennedy pathway enzymes in PC synthesis, such as CHKα [16].

Oral squamous cell carcinoma exhibits elevated CCTα expression compared to adjacent non-tumor tissue [138], with the expression level positively correlating with tumor size and lymph node metastasis.

In diffuse large B-cell lymphoma, CCTα expression surpasses that in reactive hyperplasia. Frequent MYC overexpression in diffuse large B-cell lymphoma leads to increased CCTα expression, pivotal for tumor growth, as evidenced by *in vivo* experiments [139].

CCTβ, on the other hand, displays lower expression in hepatocellular carcinoma tumors compared to healthy liver tissue [137]. Its heightened presence in the tumor is associated with improved prognoses, indicating an anti-tumor characteristic in hepatocellular carcinoma. These properties hinge on the catalytic capabilities of CCTβ, impacting lipid droplet coalescence in the liver. Consequently, this diminishes fatty acid release from organelles by hormone-sensitive lipase (HSL), leading to reduced mTORC1 activation and diminished cancer cell proliferation.

#### 3.2.2. Phosphocholine Cytidylyltransferases in Other Diseases

The synthesis of PC through the Kennedy pathway, involving CCT, is implicated in various neurological disorders. CCTs are implicated in drug dependence, with reduced CCT activity observed in the putamen of individuals addicted to cocaine (but not methamphetamine) [140]. Studies in rats reveal a decline in CCTβ expression in the Purkinje cell layer due to cocaine, bearing significant implications for addiction progression and treatment [141]. Administration of CDP-choline (citicoline), a product of this enzyme, demonstrates potential in alleviating cocaine craving [142], underscoring the significance of reduced CCTβ expression in cocaine addiction.

Patients with Parkinson’s disease exhibit elevated activity of enzymes involved in phospholipid biosynthesis (PE, PC, and phosphatidylserine (PS)) in the substantia nigra [143]. Despite no differences in the activity of other enzymes in *de novo* phospholipid synthesis between Parkinson’s disease patients and those without the condition, increased CCT activity corresponds to higher PC levels in the brains of affected individuals. Consequently, elevated PC levels in cerebrospinal fluid compared to healthy individuals suggest a compensatory mechanism for neural tissue damage during Parkinson’s disease progression [144]. The involvement of phospholipids in α-synuclein aggregation and Parkinson’s disease progression, particularly the fatty acid composition in PC, underscores the role of increased CCT activity in the substantia nigra in contributing to disease development [145,146].

CCTα’s significance extends to heart diseases, where ischemia, especially hypoxia and glucose deprivation, diminishes choline kinase and CCT activity in cardiomyocytes [147]. This results in reduced PC production through the Kennedy pathway, leading to lower levels of specific PC species in the heart during ischemia [148]. The consequent disturbance in cell membrane function leads to cardiomyocyte necrosis, emphasizing the potential therapeutic effects of normalizing PC levels in cardiomyocytes for certain heart diseases.

CCTα’s involvement in obesity is marked by a positive correlation between PCYT1A expression and body mass index (BMI) and waist-to-hip ratio in adipose tissue [45]. In obesity, adipocyte breakdown leads to the formation of crown-like structures in adipose tissue, where macrophages accumulate [149]. The released TAGs are taken up by macrophages, triggering inflammatory reactions in adipose tissue macrophages [150]. Lipids and increased PC production in these cells contribute to inflammation, involving an upregulation of enzymes in the Kennedy pathway for PC production, including CCTα and CEPT1, and the choline transporter SLC44A1 [45]. This pathway indirectly affects PC turnover, leading to decreased polyunsaturated fatty acid (PUFA) levels in macrophage membrane phospholipids, resulting in endoplasmic reticulum (ER) stress and inflammatory reactions that contribute to the consequences of obesity in adipose tissue and other organs [45].

CCTs play a role in liver diseases, with reduced CCTα activity linked to non-alcoholic steatohepatitis (NASH) [151]. This association arises from the enzyme’s involvement in VLDL secretion and hepatic fatty acid uptake. Conversely, reduced CCTβ expression in choline deficiency can inhibit the development of liver diseases, as evidenced by increased CCTβ expression in the liver. This p53-dependent process increases PC synthesis, reducing lipid droplet coalescence, ultimately protecting the liver from hepatic steatosis and positioning CCTβ as an anti-oncogene in hepatocellular carcinoma [137].

CCTα plays a pivotal role in the progression of hepatitis B virus (HBV) infection [152,153,154]. Hepatocyte infection by HBV results in an upregulation of CCTα expression, alongside other enzymes involved in PC synthesis, including CHKα and cholinephosphotransferase 1 (CHPT1) [152]. Simultaneously, there is a downregulation of phosphoethanolamine/phosphocholine phosphatase 1 (PHOSPHO1), the enzyme responsible for phosphocholine breakdown [152,153]. This molecular cascade leads to an augmented production of PC [152,153,154]. The indispensability of CCTα in HBV replication is evident, as a reduction in its expression hampers the replication of HBV [153]. Consequently, the strategic targeting of CCTα holds promise for therapeutic interventions against HBV.

### 3.3. Cholinephosphotransferase

In the third reaction of the Kennedy pathway, CDP-choline and DAG are converted into PC [2]. Enzymes catalyzing this reaction are cholinephosphotransferases. Such activity is exhibited by CEPT1 [47] and CHPT1 [155]. It is worth noting that CEPT1 also displays ethanolaminephosphotransferase activity [47,48], while CHPT1 does not [50,155]. However, the differences between these enzymes do not end there. CEPT1 is a membrane protein located in the endoplasmic reticulum and nuclear membranes [50,51], while CHPT1 is located in the Golgi apparatus [50]. CHPT1 exhibits the highest activity toward 1-palmitoyl-2-oleyl-glycerol [50], whereas CEPT1 exhibits the highest activity toward dioleyl-glycerol and dipalmitoleoylo-glycerol when CDP-choline is the substrate [48]. Increased expression of lysophosphatidylcholine acyltransferase 1 (LPCAT1) leads to ubiquitination and degradation of CEPT1 protein [156]. That is, PC production by the Kennedy pathway is reduced by PC resynthesis in Lands cycles.

#### Cholinephosphotransferase in Diseases

CHPT1’s involvement in tumorigenic processes reveals its pro-tumor properties in a breast cancer model. The expression of CHPT1 experiences a reduction in breast cancer tumors compared to healthy tissue [157]. Estrogen receptor α (ERα) heightens the expression of this enzyme, fostering cancer cell proliferation and enabling anchorage-independent growth [158]. Interestingly, heightened CHPT1 expression in breast invasive carcinoma correlates with more favorable prognoses, suggesting that this enzyme may not actively contribute to tumorigenic processes in breast cancer [14].

Conversely, lung adenocarcinoma exhibits lower CHPT1 expression than healthy lung tissue [14,16]. In this cancer type, PC synthesis through the Kennedy pathway is linked to anti-tumor effects, as higher CHKα expression, the first enzyme in PC synthesis in the Kennedy pathway, correlates with better patient outcomes for lung adenocarcinoma [16].

CHPT1 plays a significant role in prostate cancer. The expression of CHPT1 is diminished in prostate cancer tumors compared to healthy tissue [159]. Moreover, the expression of this enzyme in prostate cancer tumors increases with Gleason score and is elevated in tumors with lymph node metastasis [159,160,161]. CHPT1 expression is upregulated by AR, both in an androgen-dependent and androgen-independent manner [160]. The heightened CHPT1 expression by androgen-independent AR leads to therapy resistance to enzalutamide, a next-generation antiandrogen used in treating castration-resistant prostate cancer. Therefore, CHPT1 inhibitors or drugs targeting other enzymes in the PC synthesis pathway could potentially overcome drug resistance in castration-resistant prostate cancer.

The discussed enzyme may also be implicated in alcohol-related liver disease (ALD). Chronic alcohol consumption diminishes CHPT1 activity [162], resulting in decreased PC levels in the liver, as evidenced in rat experiments [163]. However, the precise impact of reduced PC production on the liver in ALD remains unclear.

PC biosynthesis undergoes changes during sepsis. In blood cells during sepsis, there is an increase in CHPT1 expression, leading to elevated PC production through the Kennedy pathway [164]. Serum during sepsis also exhibits increased acyl-CoA:lysophosphatidylcholine acyltransferases (LPCATs) activity, contributing to PC production in the Lands cycle [165]. Newly produced PC plays a role in sepsis, being essential for the secretion of proinflammatory cytokines such as TNF-α and interleukin-6 (IL-6), as demonstrated in macrophage experiments [166]. Consequently, these cells produce PC in response to lipopolysaccharide (LPS) [167]. However, not all tissues experience increased PC production during sepsis—in organs like the lungs [168], spleen, and thymus [169], there is reduced PC production under LPS influence. The composition of fatty acids in the spleen and thymus also changes, with a higher proportion of unsaturated fatty acids, particularly arachidonic acid C20:4n-6 [169]. This alteration is crucial in the excessive pro-inflammatory response in sepsis.

CHPT1 also participates in kidney ischemia–reperfusion injury. The expression of CHPT1 and PCYT2 decreases in this organ under ischemia–reperfusion conditions [170], leading to reduced levels of PE and PC 34:6 in the kidneys, as demonstrated in mouse studies [171]. Additionally, there is a decrease in plasmanyl-PC and plasmanyl-PE in the kidneys after ischemia–reperfusion injury. The exact role of these enzymes and changes in lipid composition in kidney ischemia–reperfusion injury is not fully understood.

## 4. Regulation of the Kennedy Pathway by SLC49A1-Mediated Choline and Ethanolamine Transport

The activity of the Kennedy pathway depends not only on the catalytic activity of its individual enzymes but also on the efficient uptake of pathway substrates. In particular, the availability of choline and ethanolamine is regulated by feline leukemia virus subgroup C cellular receptor 1 (FLVCR1), also known as SLC49A1 or MFSD7B [172,173,174]. The *FLVCR1* gene encodes two protein isoforms: FLVCR1a, which is localized to the plasma membrane, and FLVCR1b, which is localized to mitochondria [175].

Pathogenic variants in *FLVCR1* are associated with posterior column ataxia with retinitis pigmentosa (PCARP) and with severe developmental disorders characterized by developmental delay, microcephaly, brain malformations, and epilepsy [176,177]. FLVCR1 also functions as a heme exporter. Accordingly, defects in *FLVCR1* may contribute to Diamond–Blackfan anemia [175,178], consistent with its important role in erythroid precursors. Moreover, *FLVCR1* expression is induced by hypoxia in an HIF-2-dependent manner [179].

FLVCR1 has also been implicated as an oncogenic factor in hepatocellular carcinoma [180], synovial sarcoma [181], and esophageal squamous cell carcinoma [182]. In esophageal squamous cell carcinoma, its oncogenic activity has been linked, at least in part, to a physical interaction between FLVCR1 and chromosome segregation 1-like protein (CSE1L) [182].

## 5. Bioinformatics Analysis of the Role of Kennedy Pathway Enzymes in Cancer

To explore the relationship between Kennedy pathway enzymes and cancer-related processes, a bioinformatics analysis was conducted using the GEPIA portal (http://gepia.cancer-pku.cn/detail.php, accessed on 27 December 2024) [14]. GEPIA is based on raw data from The Cancer Genome Atlas (TCGA) [183]. The analysis examined the correlation between the expression levels of Kennedy pathway enzymes and overall survival (OS) in patients with 33 different cancer types. This correlation provides insight into the significance of these enzymes in tumor progression. Additionally, the roles of PEMT and PISD were analyzed. PEMT catalyzes the conversion of PE to PC [184], while PISD converts PS to PE [185], representing alternative biosynthetic routes for PE and PC production.

The association between gene expression levels and prognosis was analyzed using the GEPIA portal. For each gene, patients in the lowest and highest quartiles of expression were compared. Statistical significance was assessed directly in GEPIA using the log-rank test (Mantel–Cox test). Separate analyses were performed for each cancer type available in GEPIA and for each gene discussed in this review. No correction for multiple testing was applied. Results with *p* < 0.05 were considered statistically significant and are presented in the figures. Results with 0.05 < *p* < 0.10 were considered potentially informative and are therefore included in the tables.

The resulting survival analyses should be interpreted with caution and regarded primarily as a basis for further investigation. In particular, they are based on mRNA expression data, which may not directly reflect protein abundance or enzymatic activity. Enzyme activity may also be regulated independently of expression levels, for example through post-translational modifications. Such multiple analyses may also generate false positive results due to the lack of available correction for multiple testing. For all these reasons, the presented bioinformatics analysis is illustrative and can only be used to outline certain patterns and processes. All conclusions from this analysis should be replicated experimentally.

In 16 out of 33 cancer types, at least one of the Kennedy pathway genes was correlated with poor prognosis. In six cancers, at least one enzyme from each of the two Kennedy pathways (for PC and PE synthesis) was linked to unfavorable survival outcomes. These findings suggest that in some cancers, the biosynthesis of both phospholipids via the Kennedy pathway plays a key role in tumor progression, while in others, either PE or PC synthesis alone is more relevant (Table 1 and Table 2).

Among the analyzed cancer types, PE biosynthesis alone was significantly associated with overall survival (OS) in patients with AML and lung adenocarcinoma (LUAD). In contrast, PC biosynthesis alone was particularly relevant in bladder urothelial carcinoma (BLCA), breast invasive carcinoma (BRCA), colon adenocarcinoma (COAD), kidney chromophobe carcinoma (KICH), kidney renal clear cell carcinoma (KIRC), skin cutaneous melanoma (SKCM), stomach adenocarcinoma (STAD), and thyroid carcinoma (THCA). A third group of cancers, including adrenocortical carcinoma (ACC), brain lower-grade glioma (LGG), mesothelioma (MESO), pancreatic adenocarcinoma (PAAD), prostate adenocarcinoma (PRAD), and uveal melanoma (UVM), showed a strong association with both Kennedy pathways, indicating the importance of both PE and PC biosynthesis in their progression.

The analysis revealed that no single enzyme discussed in this review serves as a universal driver of cancer progression across a broad range of tumor types. The maximum number of cancers in which a given gene was correlated with poor prognosis was five, observed for *PCYT1A* and *SELENOI*. This suggests that in different cancers, specific steps of the Kennedy pathway are more critical for tumor progression than others.

In lung squamous cell carcinoma (LUSC) and skin cutaneous melanoma (SKCM), high *PEMT* expression was correlated with poor prognosis. Notably, enzymes involved in PE synthesis via the Kennedy pathway were not linked to poor survival in these cancers, reinforcing the idea that PC biosynthesis from PE, rather than *de novo* PC synthesis, is particularly significant. However, in skin cutaneous melanoma (SKCM), both *PEMT*-mediated PC production from PE and Kennedy pathway-mediated PC biosynthesis appear to be relevant to tumor progression.

## 6. Limitations

In this review, we linked enzymes of the Kennedy pathway to the molecular mechanisms underlying a range of diseases. It should be emphasized, however, that most of the cited studies examined the expression of these enzymes. Changes in expression alone may not necessarily reflect their functional relevance in disease processes. Enzymatic activity is likely to be more informative, as it is often regulated independently of expression levels through post-translational modifications of the enzyme protein. Therefore, the data summarized in this review should be interpreted with appropriate caution.

## Figures and Tables

**Table 1:** The impact of expression levels of enzymes responsible for PE synthesis on overall survival in patients with selected cancers.

Name of the Cancer	*ETNK1*	*ETNK2*	*PCYT2*	*CEPT1*	*SELENOI*/*EPT1*	*PISD*
Adrenocortical carcinoma	-	↑ *p* = 0.093	-	↓ *p* = 0.045	↓ *p* = 0.0037	-
Bladder urothelial carcinoma	-	-	-	-	-	↑ *p* = 0.02
Breast invasive carcinoma	-	-	↑ *p* = 0.032	-	-	-
Cervical squamous cell carcinoma and endocervical adenocarcinoma	-	-	-	-	-	-
Cholangiocarcinoma	-	-	-	-	-	-
Colon adenocarcinoma	↑ *p* = 0.059	-	-	-	-	-
Lymphoid neoplasm diffuse large B-cell lymphoma	-	-	-	-	-	-
Esophageal carcinoma	-	-	↓ *p* = 0.061	-	-	-
Glioblastoma multiforme	↑ *p* = 0.014	↓ *p* = 0.0017	-	↑ *p* = 0.062	-	-
Head and neck squamous cell carcinoma	-	-	-	-	↓ *p* = 0.079	-
Kidney chromophobe	-	-	-	-	-	-
Kidney renal clear cell carcinoma	↑ *p* = 0.015	↑ *p* = 3.1 × 10^−8^	↑ *p* = 0.085	↑ *p* = 0.0035	↑ *p* = 0.0017	↓ *p* = 0.083
Kidney renal papillary cell carcinoma	-	-	↑ *p* = 0.012	-	-	-
Acute myeloid leukemia	-	↓ *p* = 0.041	-	-	-	-
Brain lower grade glioma	↓ *p* = 0.00068	↓ *p* = 0.0011	-	↓ *p* = 2 × 10^−6^	-	-
Liver hepatocellular carcinoma	-	-	↑ *p* = 0.062	-	↓ *p* = 0.054	↓ *p* = 0.042
Lung adenocarcinoma	↓ *p* = 0.047	-	-	-	↓ *p* = 0.00073	-
Lung squamous cell carcinoma	-	-	-	-	-	-
Mesothelioma	-	-	-	-	↓ *p* = 0.014	-
Ovarian serous cystadenocarcinoma	-	-	-	-	-	-
Pancreatic adenocarcinoma	-	-	-	↓ *p* = 0.041	-	↑ *p* = 0.0056
Pheochromocytoma and Paraganglioma	-	-	-	-	-	-
Prostate adenocarcinoma	-	-	-	-	↓ *p* = 0.025	-
Rectum adenocarcinoma	-	-	-	↑ *p* = 0.047	-	↑ *p* = 0.0066
Sarcoma	-	-	↓ *p* = 0.083	-	↓ *p* = 0.078	-
Skin cutaneous melanoma	-	-	-	-	-	-
Stomach adenocarcinoma	-	-	-	-	-	-
Testicular germ cell tumors	-	-	-	-	-	-
Thyroid carcinoma	-	-	-	-	-	-
Thymoma	-	-	-	-	-	-
Uterine corpus endometrial carcinoma	-	-	-	-	-	-
Uterine carcinosarcoma	-	-	-	-	-	-
Uveal Melanoma	-	-	-	-	↓ *p* = 0.031	↓ *p* = 0.0011

↓, red background—higher expression is correlated with a worse prognosis for a patient with a given cancer; ↑, blue background—lower expression is correlated with a worse prognosis for a patient with a given cancer; -, gray background—expression of a particular gene is not significantly correlated with patient prognosis.

**Table 2:** The impact of expression levels of enzymes responsible for PC synthesis on overall survival in patients with selected cancers.

Name of the Cancer	*CHKA*	*CHKB*	*PCYT1A*	*PCYT1B*	*CEPT1*	*CHPT1*	*PEMT*
Adrenocortical carcinoma	↓ *p* = 0.00027	↓ *p* = 0.018	-	↑ *p* = 0.031	↓ *p* = 0.045	-	-
Bladder urothelial carcinoma	-	-	-	↓ *p* = 0.018	-	-	-
Breast invasive carcinoma	-	↑ *p* = 0.097	↓ *p* = 0.0073	-	-	↑ *p* = 0.0045	-
Cervical squamous cell carcinoma and endocervical adenocarcinoma	-	↑	-	-	-	-	-
Cholangiocarcinoma	-	↑ *p* = 0.099	-	-	-	↓ *p* = 0.058	↓ *p* = 0.085
Colon adenocarcinoma	-	-	-	↓ *p* = 0.022	-	-	-
Lymphoid neoplasm diffuse large B-cell lymphoma	↓ *p* = 0.051	-	-	N.A.	-	-	-
Esophageal carcinoma	-	-	-	-	-	↓ *p* = 0.072	-
Glioblastoma multiforme	-	-	-	-	↑ *p* = 0.062	-	-
Head and neck squamous cell carcinoma	-	-	-	-	-	-	-
Kidney chromophobe	-	↑ *p* = 0.093	↓ *p* = 0.043	-	-	-	-
Kidney renal clear cell carcinoma	↓ *p* = 0.070	↓ *p* = 0.012	↑ *p* = 0.042	-	↑ *p* = 0.0035	↑ *p* = 0.017	-
Kidney renal papillary cell carcinoma	-	-	-	-	-	-	-
Acute myeloid leukemia	-	-	-	-	-	-	-
Brain lower grade glioma	-	-	↓ *p* = 0.078	-	↓ *p* = 2 × 10^−06^	↓ *p* = 0.086	-
Liver hepatocellular carcinoma	↓ *p* = 0.022	-	↓ *p* = 0.032	-	-	-	-
Lung adenocarcinoma	↑ *p* = 0.0059	-	-	-	-	↓ *p* = 0.063	-
Lung squamous cell carcinoma	-	-	-	-	-	-	↓ *p* = 0.043
Mesothelioma	-	-	↓ *p* = 0.092	-	-	↓ *p* = 0.00012	↓ *p* = 0.082
Ovarian serous cystadenocarcinoma	-	-	-	-	-	-	-
Pancreatic adenocarcinoma	-	-	↓ *p* = 0.05	-	↓ *p* = 0.041	-	↑ *p* = 0.028
Pheochromocytoma and Paraganglioma	↓ *p* = 0.065	↑ *p* = 0.014	-	↓ *p* = 0.075	-	-	-
Prostate adenocarcinoma	↓ *p* = 0.055	-	↓ *p* = 0.039	-	-	-	-
Rectum adenocarcinoma	-	-	-	-	↑ *p* = 0.047	-	-
Sarcoma	-	-	-	↑ *p* = 0.023	-	-	-
Skin cutaneous melanoma	↓ *p* = 0.036	-	-	-	-	↑ *p* = 0.097	↓ *p* = 0.018
Stomach adenocarcinoma	-	-	-	↓ *p* = 0.042	-	-	-
Testicular germ cell tumors	-	-	-	-	-	-	-
Thyroid carcinoma	-	↑ *p* = 0.04	↓ *p* = 0.070	↓ *p* = 0.018	-	↑ *p* = 0.064	-
Thymoma	-	-	-	↑ *p* = 0.090	-	-	-
Uterine corpus endometrial carcinoma	-	-	-	-	-	-	-
Uterine carcinosarcoma	-	-	-	-	-	-	-
Uveal Melanoma	-	↓ *p* = 0.085	-	-	-	↓ *p* = 0.0019	-

↓, red background—higher expression is correlated with a worse prognosis for a patient with a given cancer; ↑, blue background—lower expression is correlated with a worse prognosis for a patient with a given cancer; -, gray background—expression of a particular gene is not significantly correlated with patient prognosis.

## Data Availability

No new data were created or analyzed in this study. Data sharing is not applicable to this article.

## References

[1] Morita S.-Y., Ikeda Y. (2022). Regulation of Membrane Phospholipid Biosynthesis in Mammalian Cells. Biochem. Pharmacol..

[2] Kennedy E.P., Weiss S.B. (1956). The Function of Cytidine Coenzymes in the Biosynthesis of Phospholipides. J. Biol. Chem..

[3] Coleman R.A., Mashek D.G. (2011). Mammalian triacylglycerol metabolism: Synthesis, lipolysis, and signaling. Chem. Rev..

[4] Korbecki J., Bosiacki M., Kupnicka P., Barczak K., Ziętek P., Chlubek D., Baranowska-Bosiacka I. (2024). Biochemistry and Diseases Related to the Interconversion of Phosphatidylcholine, Phosphatidylethanolamine, and Phosphatidylserine. Int. J. Mol. Sci..

[5] Lykidis A., Wang J., Karim M.A., Jackowski S. (2001). Overexpression of a Mammalian Ethanolamine-Specific Kinase Accelerates the CDP-Ethanolamine Pathway. J. Biol. Chem..

[6] Draus E., Niefind J., Vietor K., Havsteen B. (1990). Isolation and Characterization of the Human Liver Ethanolamine Kinase. Biochim. Biophys. Acta.

[7] Fagerberg L., Hallström B.M., Oksvold P., Kampf C., Djureinovic D., Odeberg J., Habuka M., Tahmasebpoor S., Danielsson A., Edlund K. (2014). Analysis of the Human Tissue-Specific Expression by Genome-Wide Integration of Transcriptomics and Antibody-Based Proteomics. Mol. Cell. Proteom. MCP.

[8] Fu G., Guy C.S., Chapman N.M., Palacios G., Wei J., Zhou P., Long L., Wang Y.-D., Qian C., Dhungana Y. (2021). Metabolic Control of TFH Cells and Humoral Immunity by Phosphatidylethanolamine. Nature.

[9] Fontana D., Mauri M., Renso R., Docci M., Crespiatico I., Røst L.M., Jang M., Niro A., D’Aliberti D., Massimino L. (2020). ETNK1 Mutations Induce a Mutator Phenotype That Can Be Reverted with Phosphoethanolamine. Nat. Commun..

[10] Shang J., Wang Q., Wang J., Xu B., Liu S. (2023). miR-708-3p Promotes Gastric Cancer Progression through Downregulating ETNK1. Heliyon.

[11] Li L., Mou Y.-P., Wang Y.-Y., Wang H.-J., Mou X.-Z. (2019). miR-199a-3p Targets ETNK1 to Promote Invasion and Migration in Gastric Cancer Cells and Is Associated with Poor Prognosis. Pathol. Res. Pract..

[12] Miwa T., Kanda M., Shimizu D., Umeda S., Sawaki K., Tanaka H., Tanaka C., Hattori N., Hayashi M., Yamada S. (2021). Hepatic Metastasis of Gastric Cancer Is Associated with Enhanced Expression of Ethanolamine Kinase 2 via the P53-Bcl-2 Intrinsic Apoptosis Pathway. Br. J. Cancer.

[13] Lou J., Liu X., Fan X., Xu X., Wang Z., Wang L. (2023). Lncrna FEZf1-As1 Negatively Regulates ETNK1 to Promote Malignant Progression of Renal Cell Carcinoma. J. Med. Biochem..

[14] Tang Z., Li C., Kang B., Gao G., Li C., Zhang Z. (2017). GEPIA: A Web Server for Cancer and Normal Gene Expression Profiling and Interactive Analyses. Nucleic Acids Res..

[15] Chu J., Qian X.-X., Zhang X.-M., Jiang T., Li X.-J., Sun W. (2023). ETNK2 Low-Expression Predicts Poor Prognosis in Renal Cell Carcinoma with Immunosuppressive Tumor Microenvironment. J. Oncol..

[16] Lesko J., Triebl A., Stacher-Priehse E., Fink-Neuböck N., Lindenmann J., Smolle-Jüttner F.-M., Köfeler H.C., Hrzenjak A., Olschewski H., Leithner K. (2021). Phospholipid Dynamics in Ex Vivo Lung Cancer and Normal Lung Explants. Exp. Mol. Med..

[17] Zheng D., Jin L., Chen B., Qi Y., Bhandari A., Wen J., Lin B., Zhang X., Zhang W. (2022). The ETNK2 Gene Promotes Progression of Papillary Thyroid Carcinoma through the HIPPO Pathway. J. Cancer.

[18] Shuai W., Zuo Z., Li N., Garces S., Jelloul F.Z., Ok C.Y., Li S., Xu J., You M.J., Wang W. (2023). ETNK1 Mutation Occurs in a Wide Spectrum of Myeloid Neoplasms and Is Not Specific for Atypical Chronic Myeloid Leukemia. Cancer.

[19] Fontana D., Ramazzotti D., Aroldi A., Redaelli S., Magistroni V., Pirola A., Niro A., Massimino L., Mastini C., Brambilla V. (2020). Integrated Genomic, Functional, and Prognostic Characterization of Atypical Chronic Myeloid Leukemia. HemaSphere.

[20] Jussila A.R., Haensel D., Gaddam S., Oro A.E. (2024). Acquisition of Drug Resistance in Basal Cell Nevus Syndrome Tumors through Basal to Squamous Cell Carcinoma Transition. J. Investig. Dermatol..

[21] Nakashima A., Hosaka K., Nikawa J. (1997). Cloning of a Human cDNA for CTP-Phosphoethanolamine Cytidylyltransferase by Complementation in vivo of a Yeast Mutant. J. Biol. Chem..

[22] Tian S., Ohtsuka J., Wang S., Nagata K., Tanokura M., Ohta A., Horiuchi H., Fukuda R. (2014). Human CTP:Phosphoethanolamine Cytidylyltransferase: Enzymatic Properties and Unequal Catalytic Roles of CTP-Binding Motifs in Two Cytidylyltransferase Domains. Biochem. Biophys. Res. Commun..

[23] Vermeulen P.S., Geelen M.J., van Golde L.M. (1994). Substrate Specificity of CTP: Phosphoethanolamine Cytidylyltransferase Purified from Rat Liver. Biochim. Biophys. Acta.

[24] Vermeulen P.S., Tijburg L.B., Geelen M.J., van Golde L.M. (1993). Immunological characterization, lipid dependence, and subcellular localization of CTP:phosphoethanolamine cytidylyltransferase purified from rat liver. Comparison with CTP: Phosphocholine cytidylyltransferase. J. Biol. Chem..

[25] van Hellemond J.J., Slot J.W., Geelen M.J., van Golde L.M., Vermeulen P.S. (1994). Ultrastructural Localization of CTP:phosphoethanolamine Cytidylyltransferase in Rat Liver. J. Biol. Chem..

[26] Tie A., Bakovic M. (2007). Alternative Splicing of CTP:Phosphoethanolamine Cytidylyltransferase Produces Two Isoforms That Differ in Catalytic Properties. J. Lipid Res..

[27] Pavlovic Z., Singh R.K., Bakovic M. (2014). A Novel Murine CTP: Phosphoethanolamine Cytidylyltransferase Splice Variant Is a Post-Translational Repressor and an Indicator That Both Cytidylyltransferase Domains Are Required for Activity. Gene.

[28] Pavlovic Z., Zhu L., Pereira L., Singh R.K., Cornell R.B., Bakovic M. (2014). Isoform-Specific and Protein Kinase C-Mediated Regulation of CTP:Phosphoethanolamine Cytidylyltransferase Phosphorylation. J. Biol. Chem..

[29] Zhu L., Michel V., Bakovic M. (2009). Regulation of the Mouse CTP: Phosphoethanolamine Cytidylyltransferase Gene Pcyt2 during Myogenesis. Gene.

[30] Cikes D., Elsayad K., Sezgin E., Koitai E., Torma F., Orthofer M., Yarwood R., Heinz L.X., Sedlyarov V., Miranda N.D. (2023). PCYT2-Regulated Lipid Biosynthesis Is Critical to Muscle Health and Ageing. Nat. Metab..

[31] Vaz F.M., McDermott J.H., Alders M., Wortmann S.B., Kölker S., Pras-Raves M.L., Vervaart M.A.T., van Lenthe H., Luyf A.C.M., Elfrink H.L. (2019). Mutations in PCYT2 Disrupt Etherlipid Biosynthesis and Cause a Complex Hereditary Spastic Paraplegia. Brain J. Neurol..

[32] Leonardis L., Skrjanec Pusenjak M., Maver A., Jaklic H., Ozura Brecko A., Koritnik B., Peterlin B., Writzl K. (2022). Axonal Polyneuropathy in 2 Brothers with a Homozygous Missense Variant in the First Catalytic Domain of PCYT2. Neurol. Genet..

[33] Osawa T., Shimamura T., Saito K., Hasegawa Y., Ishii N., Nishida M., Ando R., Kondo A., Anwar M., Tsuchida R. (2019). Phosphoethanolamine Accumulation Protects Cancer Cells under Glutamine Starvation through Downregulation of PCYT2. Cell Rep..

[34] Imae R., Manya H., Tsumoto H., Miura Y., Endo T. (2021). PCYT2 Synthesizes CDP-Glycerol in Mammals and Reduced PCYT2 Enhances the Expression of Functionally Glycosylated α-Dystroglycan. J. Biochem..

[35] Imae R., Manya H., Tsumoto H., Osumi K., Tanaka T., Mizuno M., Kanagawa M., Kobayashi K., Toda T., Endo T. (2018). CDP-Glycerol Inhibits the Synthesis of the Functional O-Mannosyl Glycan of α-Dystroglycan. J. Biol. Chem..

[36] Umezawa F., Natsume M., Fukusada S., Nakajima K., Yamasaki F., Kawashima H., Kuo C.-W., Khoo K.-H., Shimura T., Yagi H. (2022). Cancer Malignancy Is Correlated with Upregulation of PCYT2-Mediated Glycerol Phosphate Modification of α-Dystroglycan. Int. J. Mol. Sci..

[37] Gohil V.M., Zhu L., Baker C.D., Cracan V., Yaseen A., Jain M., Clish C.B., Brookes P.S., Bakovic M., Mootha V.K. (2013). Meclizine Inhibits Mitochondrial Respiration through Direct Targeting of Cytosolic Phosphoethanolamine Metabolism. J. Biol. Chem..

[38] Wang Z., Lee B., Pearce D., Qian S., Wang Y., Zhang Q., Chow M.S.S. (2012). Meclizine Metabolism and Pharmacokinetics: Formulation on Its Absorption. J. Clin. Pharmacol..

[39] Guan Y., Chen X., Wu M., Zhu W., Arslan A., Takeda S., Nguyen M.H., Majeti R., Thomas D., Zheng M. (2020). The Phosphatidylethanolamine Biosynthesis Pathway Provides a New Target for Cancer Chemotherapy. J. Hepatol..

[40] Victor P.P., Narayanaswamy R., Kadry S., Gurunathan B. (2023). Identification of Novel Inhibitor against Human Phosphoethanolamine Cytidylyltransferase from Phytochemicals of Citrus Sinensis Peel Extract by In Vitro and In Silico Approach. Biotechnol. Appl. Biochem..

[41] Xu H., Li W., Huang L., He X., Xu B., He X., Chen W., Wang Y., Xu W., Wang S. (2023). Phosphoethanolamine Cytidylyltransferase Ameliorates Mitochondrial Function and Apoptosis in Hepatocytes in T2DM In Vitro. J. Lipid Res..

[42] Fullerton M.D., Hakimuddin F., Bakovic M. (2007). Developmental and Metabolic Effects of Disruption of the Mouse CTP: Phosphoethanolamine Cytidylyltransferase Gene (Pcyt2). Mol. Cell. Biol..

[43] Fullerton M.D., Bakovic M. (2010). Complementation of the Metabolic Defect in CTP:Phosphoethanolamine Cytidylyltransferase (*Pcyt2*)–Deficient Primary Hepatocytes. Metabolism.

[44] Basu P., Alibhai F.J., Tsimakouridze E.V., Singh R.K., Paglialunga S., Holloway G.P., Martino T.A., Bakovic M. (2015). Male-Specific Cardiac Dysfunction in CTP:Phosphoethanolamine Cytidylyltransferase (Pcyt2)-Deficient Mice. Mol. Cell. Biol..

[45] Sharma N.K., Langberg K.A., Mondal A.K., Das S.K. (2013). Phospholipid Biosynthesis Genes and Susceptibility to Obesity: Analysis of Expression and Polymorphisms. PLoS ONE.

[46] Grapentine S., Singh R.K., Basu P., Sivanesan S., Mattos G., Oresajo O., Cheema J., Demeke W., Dolinsky V.W., Bakovic M. (2022). Pcyt2 Deficiency Causes Age-Dependant Development of Nonalcoholic Steatohepatitis and Insulin Resistance That Could Be Attenuated with Phosphoethanolamine. Sci. Rep..

[47] Henneberry A.L., McMaster C.R. (1999). Cloning and Expression of a Human Choline/Ethanolaminephosphotransferase: Synthesis of Phosphatidylcholine and Phosphatidylethanolamine. Biochem. J..

[48] Wright M.M., McMaster C.R. (2002). PC and PE Synthesis: Mixed Micellar Analysis of the Cholinephosphotransferase and Ethanolaminephosphotransferase Activities of Human Choline/Ethanolamine Phosphotransferase 1 (CEPT1). Lipids.

[49] Horibata Y., Hirabayashi Y. (2007). Identification and Characterization of Human Ethanolaminephosphotransferase1. J. Lipid Res..

[50] Henneberry A.L., Wright M.M., McMaster C.R. (2002). The Major Sites of Cellular Phospholipid Synthesis and Molecular Determinants of Fatty Acid and Lipid Head Group Specificity. Mol. Biol. Cell.

[51] Horibata Y., Ando H., Sugimoto H. (2020). Locations and Contributions of the Phosphotransferases EPT1 and CEPT1 to the Biosynthesis of Ethanolamine Phospholipids. J. Lipid Res..

[52] Horibata Y., Elpeleg O., Eran A., Hirabayashi Y., Savitzki D., Tal G., Mandel H., Sugimoto H. (2018). EPT1 (Selenoprotein I) Is Critical for the Neural Development and Maintenance of Plasmalogen in Humans. J. Lipid Res..

[53] Nunes L.G.A., Pitts M.W., Hoffmann P.R. (2022). Selenoprotein I (Selenoi) as a Critical Enzyme in the Central Nervous System. Arch. Biochem. Biophys..

[54] Ahmed M.Y., Al-Khayat A., Al-Murshedi F., Al-Futaisi A., Chioza B.A., Pedro Fernandez-Murray J., Self J.E., Salter C.G., Harlalka G.V., Rawlins L.E. (2017). A Mutation of EPT1 (SELENOI) Underlies a New Disorder of Kennedy Pathway Phospholipid Biosynthesis. Brain J. Neurol..

[55] Ma C., Hoffmann F.W., Marciel M.P., Page K.E., Williams-Aduja M.A., Akana E.N.L., Gojanovich G.S., Gerschenson M., Urschitz J., Moisyadi S. (2021). Upregulated Ethanolamine Phospholipid Synthesis via Selenoprotein I Is Required for Effective Metabolic Reprogramming during T Cell Activation. Mol. Metab..

[56] Ma C., Hoffmann F.W., Nunes L.G., Urena F., Andrukhiv A., Gerschenson M., Pitts M.W., Hoffmann P.R. (2022). Selenoprotein I Deficiency in T Cells Promotes Differentiation into Tolerant Phenotypes While Decreasing Th17 Pathology. J. Leukoc. Biol..

[57] Barlin J.N., Jelinic P., Olvera N., Bogomolniy F., Bisogna M., Dao F., Barakat R.R., Chi D.S., Levine D.A. (2013). Validated Gene Targets Associated with Curatively Treated Advanced Serous Ovarian Carcinoma. Gynecol. Oncol..

[58] Zekri A.-R.N., Hassan Z.K., Bahnassy A.A., Eldahshan D.H., El-Rouby M.N.E., Kamel M.M., Hafez M.M. (2013). Gene Expression Profiling of Non-Hodgkin Lymphomas. Asian Pac. J. Cancer Prev. APJCP.

[59] Ecker A., Barbosa N.V., Ardisson-Araujo D. (2020). Accessing the Transcriptional Status of Selenoproteins in Skin Cancer-Derived Cell Lines. J. Trace Elem. Med. Biol. Organ Soc. Miner. Trace Elem..

[60] Funai K., Lodhi I.J., Spears L.D., Yin L., Song H., Klein S., Semenkovich C.F. (2016). Skeletal Muscle Phospholipid Metabolism Regulates Insulin Sensitivity and Contractile Function. Diabetes.

[61] Henne S.K., Aldisi R., Sivalingam S., Hochfeld L.M., Borisov O., Krawitz P.M., Maj C., Nöthen M.M., Heilmann-Heimbach S. (2023). Analysis of 72,469 UK Biobank Exomes Links Rare Variants to Male-Pattern Hair Loss. Nat. Commun..

[62] Korbecki J., Bosiacki M., Kupnicka P., Barczak K., Ziętek P., Chlubek D., Baranowska-Bosiacka I. (2025). Choline Kinases: Enzymatic Activity, Involvement in Cancer and Other Diseases, Inhibitors. Int. J. Cancer.

[63] Aoyama C., Yamazaki N., Terada H., Ishidate K. (2000). Structure and Characterization of the Genes for Murine Choline/Ethanolamine Kinase Isozymes α and Β1. J. Lipid Res..

[64] Gallego-Ortega D., Ramirez de Molina A., Ramos M.A., Valdes-Mora F., Barderas M.G., Sarmentero-Estrada J., Lacal J.C. (2009). Differential Role of Human Choline Kinase Alpha and Beta Enzymes in Lipid Metabolism: Implications in Cancer Onset and Treatment. PLoS ONE.

[65] Uchida T. (1994). Regulation of Choline Kinase R: Analyses of Alternatively Spliced Choline Kinases and the Promoter Region. J. Biochem..

[66] Miyake T., Parsons S.J. (2012). Functional Interactions between Choline Kinase α, Epidermal Growth Factor Receptor and c-Src in Breast Cancer Cell Proliferation. Oncogene.

[67] Chang C.C., Few L.L., Konrad M., See Too W.C. (2016). Phosphorylation of Human Choline Kinase Beta by Protein Kinase A: Its Impact on Activity and Inhibition. PLoS ONE.

[68] Buchser W.J., Slepak T.I., Gutierrez-Arenas O., Bixby J.L., Lemmon V.P. (2010). Kinase/Phosphatase Overexpression Reveals Pathways Regulating Hippocampal Neuron Morphology. Mol. Syst. Biol..

[69] Myers A.J., Potts C., Makarewicz J.A., McGee E., Dumas J.A. (2024). Choline Kinase Alpha Genotype Is Related to Hippocampal Brain Volume and Cognition in Postmenopausal Women. Heliyon.

[70] Klöckner C., Fernández-Murray J.P., Tavasoli M., Sticht H., Stoltenburg-Didinger G., Scholle L.M., Bakhtiari S., Kruer M.C., Darvish H., Firouzabadi S.G. (2022). Bi-Allelic Variants in CHKA Cause a Neurodevelopmental Disorder with Epilepsy and Microcephaly. Brain J. Neurol..

[71] Kular J., Tickner J.C., Pavlos N.J., Viola H.M., Abel T., Lim B.S., Yang X., Chen H., Cook R., Hool L.C. (2015). Choline Kinase β Mutant Mice Exhibit Reduced Phosphocholine, Elevated Osteoclast Activity, and Low Bone Mass. J. Biol. Chem..

[72] Bardhan M., Polavarapu K., Bevinahalli N.N., Veeramani P.-K., Anjanappa R.M., Arunachal G., Shingavi L., Vengalil S., Nashi S., Chawla T. (2021). Megaconial Congenital Muscular Dystrophy Secondary to Novel CHKB Mutations Resemble Atypical Rett Syndrome. J. Hum. Genet..

[73] Zemorshidi F., Nafissi S., Boostani R., Karimiani E.G., Ashtiani B.H., Karimzadeh P., Miryounesi M., Tonekaboni S.H., Nilipour Y. (2023). Megaconial Congenital Muscular Dystrophy Due to CHKB Gene Variants, the First Report of Thirteen Iranian Patients. Neuromuscul. Disord..

[74] Ma W., Wang S., Zhang T., Zhang E.Y., Zhou L., Hu C., Yu J.J., Xu G. (2018). Activation of Choline Kinase Drives Aberrant Choline Metabolism in Esophageal Squamous Cell Carcinomas. J. Pharm. Biomed. Anal..

[75] Kwee S.A., Hernandez B., Chan O., Wong L. (2012). Choline Kinase Alpha and Hexokinase-2 Protein Expression in Hepatocellular Carcinoma: Association with Survival. PLoS ONE.

[76] Ramírez de Molina A., Sarmentero-Estrada J., Belda-Iniesta C., Tarón M., Ramírez de Molina V., Cejas P., Skrzypski M., Gallego-Ortega D., de Castro J., Casado E. (2007). Expression of Choline Kinase Alpha to Predict Outcome in Patients with Early-Stage Non-Small-Cell Lung Cancer: A Retrospective Study. Lancet Oncol..

[77] Challapalli A., Trousil S., Hazell S., Kozlowski K., Gudi M., Aboagye E.O., Mangar S. (2015). Exploiting Altered Patterns of Choline Kinase-Alpha Expression on Human Prostate Tissue to Prognosticate Prostate Cancer. J. Clin. Pathol..

[78] Ramírez de Molina A., Rodríguez-González A., Penalva V., Lucas L., Lacal J.C. (2001). Inhibition of ChoK Is an Efficient Antitumor Strategy for Harvey-, Kirsten-, and N-Ras-Transformed Cells. Biochem. Biophys. Res. Commun..

[79] Ramírez de Molina A., Gutiérrez R., Ramos M.A., Silva J.M., Silva J., Bonilla F., Sánchez J.J., Lacal J.C. (2002). Increased Choline Kinase Activity in Human Breast Carcinomas: Clinical Evidence for a Potential Novel Antitumor Strategy. Oncogene.

[80] Hunt A.N., Clark G.T., Attard G.S., Postle A.D. (2001). Highly Saturated Endonuclear Phosphatidylcholine Is Synthesizedin Situ and Colocated with CDP-Choline Pathway Enzymes. J. Biol. Chem..

[81] Gruber J., See Too W.C., Wong M.T., Lavie A., McSorley T., Konrad M. (2012). Balance of Human Choline Kinase Isoforms Is Critical for Cell Cycle Regulation: Implications for the Development of Choline Kinase-Targeted Cancer Therapy. FEBS J..

[82] Lin X.-M., Hu L., Gu J., Wang R.-Y., Li L., Tang J., Zhang B.-H., Yan X.-Z., Zhu Y.-J., Hu C.-L. (2017). Choline Kinase α Mediates Interactions Between the Epidermal Growth Factor Receptor and Mechanistic Target of Rapamycin Complex 2 in Hepatocellular Carcinoma Cells to Promote Drug Resistance and Xenograft Tumor Progression. Gastroenterology.

[83] Chua B.T., Gallego-Ortega D., Ramirez de Molina A., Ullrich A., Lacal J.C., Downward J. (2009). Regulation of Akt(Ser473) Phosphorylation by Choline Kinase in Breast Carcinoma Cells. Mol. Cancer.

[84] Asim M., Massie C.E., Orafidiya F., Pértega-Gomes N., Warren A.Y., Esmaeili M., Selth L.A., Zecchini H.I., Luko K., Qureshi A. (2016). Choline Kinase Alpha as an Androgen Receptor Chaperone and Prostate Cancer Therapeutic Target. J. Natl. Cancer Inst..

[85] Mariotto E., Bortolozzi R., Volpin I., Carta D., Serafin V., Accordi B., Basso G., Navarro P.L., López-Cara L.C., Viola G. (2018). EB-3D a Novel Choline Kinase Inhibitor Induces Deregulation of the AMPK-mTOR Pathway and Apoptosis in Leukemia T-Cells. Biochem. Pharmacol..

[86] Lacal J.C., Perona R., de Castro J., Cebrián A. (2022). Choline Kinase α Inhibitors MN58b and RSM932A Enhances the Antitumor Response to Cisplatin in Lung Tumor Cells. Pharmaceutics.

[87] Wang N., Brickute D., Braga M., Barnes C., Lu H., Allott L., Aboagye E.O. (2021). Novel Non-Congeneric Derivatives of the Choline Kinase Alpha Inhibitor ICL-CCIC-0019. Pharmaceutics.

[88] García-Molina P., Sola-Leyva A., Luque-Navarro P.M., Laso A., Ríos-Marco P., Ríos A., Lanari D., Torretta A., Parisini E., López-Cara L.C. (2022). Anticancer Activity of the Choline Kinase Inhibitor PL48 Is Due to Selective Disruption of Choline Metabolism and Transport Systems in Cancer Cell Lines. Pharmaceutics.

[89] Zech S.G., Kohlmann A., Zhou T., Li F., Squillace R.M., Parillon L.E., Greenfield M.T., Miller D.P., Qi J., Thomas R.M. (2016). Novel Small Molecule Inhibitors of Choline Kinase Identified by Fragment-Based Drug Discovery. J. Med. Chem..

[90] Schiaffino-Ortega S., Baglioni E., Mariotto E., Bortolozzi R., Serrán-Aguilera L., Ríos-Marco P., Carrasco-Jimenez M.P., Gallo M.A., Hurtado-Guerrero R., Marco C. (2016). Design, Synthesis, Crystallization and Biological Evaluation of New Symmetrical Biscationic Compounds as Selective Inhibitors of Human Choline Kinase A1 (ChoKα1). Sci. Rep..

[91] Schiaffino-Ortega S., López-Cara L.C., Ríos-Marco P., Carrasco-Jimenez M.P., Gallo M.A., Espinosa A., Marco C., Entrena A. (2013). New Non-Symmetrical Choline Kinase Inhibitors. Bioorganic Med. Chem..

[92] Kalmar G.B., Kay R.J., LaChance A.C., Cornell R.B. (1994). Primary Structure and Expression of a Human CTP: Phosphocholine Cytidylyltransferase. Biochim. Biophys. Acta BBA-Gene Struct. Expr..

[93] Lykidis A., Murti K.G., Jackowski S. (1998). Cloning and Characterization of a Second Human CTP:Phosphocholine Cytidylyltransferase. J. Biol. Chem..

[94] Friesen J.A., Campbell H.A., Kent C. (1999). Enzymatic and Cellular Characterization of a Catalytic Fragment of CTP:Phosphocholine Cytidylyltransferase A. J. Biol. Chem..

[95] Lee J., Taneva S.G., Holland B.W., Tieleman D.P., Cornell R.B. (2014). Structural Basis for Autoinhibition of CTP:Phosphocholine Cytidylyltransferase (CCT), the Regulatory Enzyme in Phosphatidylcholine Synthesis, by Its Membrane-Binding Amphipathic Helix. J. Biol. Chem..

[96] Ramezanpour M., Lee J., Taneva S.G., Tieleman D.P., Cornell R.B. (2018). An Auto-Inhibitory Helix in CTP:Phosphocholine Cytidylyltransferase Hijacks the Catalytic Residue and Constrains a Pliable, Domain-Bridging Helix Pair. J. Biol. Chem..

[97] Davies S.M., Epand R.M., Kraayenhof R., Cornell R.B. (2001). Regulation of CTP: Phosphocholine Cytidylyltransferase Activity by the Physical Properties of Lipid Membranes: An Important Role for Stored Curvature Strain Energy. Biochemistry.

[98] Dennis M.K., Taneva S.G., Cornell R.B. (2011). The Intrinsically Disordered Nuclear Localization Signal and Phosphorylation Segments Distinguish the Membrane Affinity of Two Cytidylyltransferase Isoforms. J. Biol. Chem..

[99] Yue L., McPhee M.J., Gonzalez K., Charman M., Lee J., Thompson J., Winkler D.F.H., Cornell R.B., Pelech S., Ridgway N.D. (2020). Differential Dephosphorylation of CTP:Phosphocholine Cytidylyltransferase upon Translocation to Nuclear Membranes and Lipid Droplets. Mol. Biol. Cell.

[100] Ryan A.J., Fisher K., Thomas C.P., Mallampalli R.K. (2004). Transcriptional Repression of the CTP:Phosphocholine Cytidylyltransferase Gene by Sphingosine. Biochem. J..

[101] Awasthi S., Vivekananda J., Awasthi V., Smith D., King R.J. (2001). CTP:Phosphocholine Cytidylyltransferase Inhibition by Ceramide via PKC-Alpha, P38 MAPK, cPLA2, and 5-Lipoxygenase. Am. J. Physiol. Lung Cell. Mol. Physiol..

[102] Bodennec J., Pelled D., Riebeling C., Trajkovic S., Futerman A.H. (2002). Phosphatidylcholine Synthesis Is Elevated in Neuronal Models of Gaucher Disease Due to Direct Activation of CTP:Phosphocholine Cytidylyltransferase by Glucosylceramide. FASEB J..

[103] Lykidis A., Baburina I., Jackowski S. (1999). Distribution of CTP:Phosphocholine Cytidylyltransferase (CCT) Isoforms: Identification of a New CCTβ Splice Variant. J. Biol. Chem..

[104] Chen B.B., Mallampalli R.K. (2009). Masking of a Nuclear Signal Motif by Monoubiquitination Leads to Mislocalization and Degradation of the Regulatory Enzyme Cytidylyltransferase. Mol. Cell. Biol..

[105] Chen B.B., Mallampalli R.K. (2007). Calmodulin Binds and Stabilizes the Regulatory Enzyme, CTP: Phosphocholine Cytidylyltransferase. J. Biol. Chem..

[106] Mallampalli R.K., Ryan A.J., Salome R.G., Jackowski S. (2000). Tumor Necrosis Factor-Alpha Inhibits Expression of CTP: Phosphocholine Cytidylyltransferase. J. Biol. Chem..

[107] Northwood I.C., Tong A.H., Crawford B., Drobnies A.E., Cornell R.B. (1999). Shuttling of CTP:Phosphocholine Cytidylyltransferase between the Nucleus and Endoplasmic Reticulum Accompanies the Wave of Phosphatidylcholine Synthesis during the G(0) → G(1) Transition. J. Biol. Chem..

[108] Golfman L.S., Bakovic M., Vance D.E. (2001). Transcription of the CTP:Phosphocholine Cytidylyltransferase Alpha Gene Is Enhanced during the S Phase of the Cell Cycle. J. Biol. Chem..

[109] Elena C., Banchio C. (2010). Specific Interaction between E2F1 and Sp1 Regulates the Expression of Murine CTP:Phosphocholine Cytidylyltransferase Alpha during the S Phase. Biochim. Biophys. Acta BBA-Mol. Cell Biol. Lipids.

[110] Lagace T.A., Ridgway N.D. (2005). The Rate-Limiting Enzyme in Phosphatidylcholine Synthesis Regulates Proliferation of the Nucleoplasmic Reticulum. Mol. Biol. Cell.

[111] Gehrig K., Cornell R.B., Ridgway N.D. (2008). Expansion of the Nucleoplasmic Reticulum Requires the Coordinated Activity of Lamins and CTP:Phosphocholine Cytidylyltransferase α. Mol. Biol. Cell.

[112] Arsenault D.J., Yoo B.H., Rosen K.V., Ridgway N.D. (2013). Ras-Induced up-Regulation of CTP:Phosphocholine Cytidylyltransferase α Contributes to Malignant Transformation of Intestinal Epithelial Cells. J. Biol. Chem..

[113] Lagace T.A., Miller J.R., Ridgway N.D. (2002). Caspase Processing and Nuclear Export of CTP:Phosphocholine Cytidylyltransferase α during Farnesol-Induced Apoptosis. Mol. Cell. Biol..

[114] Morton C.C., Aitchison A.J., Gehrig K., Ridgway N.D. (2013). A Mechanism for Suppression of the CDP-Choline Pathway during Apoptosis. J. Lipid Res..

[115] Karim M., Jackson P., Jackowski S. (2003). Gene Structure, Expression and Identification of a New CTP:Phosphocholine Cytidylyltransferase Beta Isoform. Biochim. Biophys. Acta.

[116] Andrejeva G., Gowan S., Lin G., Wong Te Fong A.-C.L., Shamsaei E., Parkes H.G., Mui J., Raynaud F.I., Asad Y., Vizcay-Barrena G. (2020). De Novo Phosphatidylcholine Synthesis Is Required for Autophagosome Membrane Formation and Maintenance during Autophagy. Autophagy.

[117] Jacobs R.L., Devlin C., Tabas I., Vance D.E. (2004). Targeted Deletion of Hepatic CTP:Phosphocholine Cytidylyltransferase Alpha in Mice Decreases Plasma High Density and Very Low Density Lipoproteins. J. Biol. Chem..

[118] Fagone P., Gunter C., Sage C.R., Gunn K.E., Brewer J.W., Jackowski S. (2009). CTP:Phosphocholine Cytidylyltransferase Alpha Is Required for B-Cell Proliferation and Class Switch Recombination. J. Biol. Chem..

[119] Kennelly J.P., van der Veen J.N., Nelson R.C., Leonard K.-A., Havinga R., Buteau J., Kuipers F., Jacobs R.L. (2018). Intestinal de Novo Phosphatidylcholine Synthesis Is Required for Dietary Lipid Absorption and Metabolic Homeostasis. J. Lipid Res..

[120] Carlin S., Kennelly J.P., Fedoruk H., Quiroga A., Leonard K.-A., Nelson R., Thiesen A., Buteau J., Lehner R., Jacobs R. (2022). De Novo Phosphatidylcholine Synthesis in the Small Intestinal Epithelium Is Required for Normal Dietary Lipid Handling and Maintenance of the Mucosal Barrier. Biochim. Biophys. Acta BBA-Mol. Cell Biol. Lipids.

[121] Enaw J.O.E., Zhu H., Yang W., Lu W., Shaw G.M., Lammer E.J., Finnell R.H. (2006). CHKA and PCYT1A Gene Polymorphisms, Choline Intake and Spina Bifida Risk in a California Population. BMC Med..

[122] Payne F., Lim K., Girousse A., Brown R.J., Kory N., Robbins A., Xue Y., Sleigh A., Cochran E., Adams C. (2014). Mutations Disrupting the Kennedy Phosphatidylcholine Pathway in Humans with Congenital Lipodystrophy and Fatty Liver Disease. Proc. Natl. Acad. Sci. USA.

[123] Hoover-Fong J., Sobreira N., Jurgens J., Modaff P., Blout C., Moser A., Kim O.-H., Cho T.-J., Cho S.Y., Kim S.J. (2014). Mutations in PCYT1A, Encoding a Key Regulator of Phosphatidylcholine Metabolism, Cause Spondylometaphyseal Dysplasia with Cone-Rod Dystrophy. Am. J. Hum. Genet..

[124] Yamamoto G.L., Baratela W.A.R., Almeida T.F., Lazar M., Afonso C.L., Oyamada M.K., Suzuki L., Oliveira L.A.N., Ramos E.S., Kim C.A. (2014). Mutations in PCYT1A Cause Spondylometaphyseal Dysplasia with Cone-Rod Dystrophy. Am. J. Hum. Genet..

[125] Testa F., Filippelli M., Brunetti-Pierri R., Di Fruscio G., Di Iorio V., Pizzo M., Torella A., Barillari M.R., Nigro V., Brunetti-Pierri N. (2017). Mutations in the PCYT1A Gene Are Responsible for Isolated Forms of Retinal Dystrophy. Eur. J. Hum. Genet..

[126] Ogasawara Y., Cheng J., Tatematsu T., Uchida M., Murase O., Yoshikawa S., Ohsaki Y., Fujimoto T. (2020). Long-Term Autophagy Is Sustained by Activation of CCTβ3 on Lipid Droplets. Nat. Commun..

[127] Strakova J., Demizieux L., Campenot R.B., Vance D.E., Vance J.E. (2011). Involvement of CTP:Phosphocholine Cytidylyltransferase-Β2 in Axonal Phosphatidylcholine Synthesis and Branching of Neurons. Biochim. Biophys. Acta.

[128] Carter J.M., Demizieux L., Campenot R.B., Vance D.E., Vance J.E. (2008). Phosphatidylcholine Biosynthesis via CTP:Phosphocholine Cytidylyltransferase 2 Facilitates Neurite Outgrowth and Branching. J. Biol. Chem..

[129] Shen Y.-J., DeLong C.J., Tercé F., Kute T., Willingham M.C., Pettenati M.J., Cui Z. (2005). Polyploid Formation via Chromosome Duplication Induced by CTP:Phosphocholine Cytidylyltransferase Deficiency and Bcl-2 Overexpression: Identification of Two Novel Endogenous Factors. J. Histochem. Cytochem. Off. J. Histochem. Soc..

[130] Fujiwara T., Bandi M., Nitta M., Ivanova E.V., Bronson R.T., Pellman D. (2005). Cytokinesis Failure Generating Tetraploids Promotes Tumorigenesis in P53-Null Cells. Nature.

[131] Storchova Z., Kuffer C. (2008). The Consequences of Tetraploidy and Aneuploidy. J. Cell Sci..

[132] Bakovic M., Waite K., Vance D.E. (2003). Oncogenic Ha-Ras Transformation Modulates the Transcription of the CTP:Phosphocholine Cytidylyltransferase Alpha Gene via P42/44MAPK and Transcription Factor Sp3. J. Biol. Chem..

[133] Momchilova A., Markovska T., Pankov R. (1999). Ha-Ras-Transformation Alters the Metabolism of Phosphatidylethanolamine and Phosphatidylcholine in NIH 3T3 Fibroblasts. Cell Biol. Int..

[134] Hu Y., Chen C., Tong X., Chen S., Hu X., Pan B., Sun X., Chen Z., Shi X., Hu Y. (2021). NSUN2 Modified by SUMO-2/3 Promotes Gastric Cancer Progression and Regulates mRNA m5C Methylation. Cell Death Dis..

[135] Hemdan T., Turker P., Malmström P.-U., Segersten U. (2018). Choline-Phosphate Cytidylyltransferase-α as a Possible Predictor of Survival and Response to Cisplatin Neoadjuvant Chemotherapy in Urothelial Cancer of the Bladder. Scand. J. Urol..

[136] Yu J., Wu C., Wu Q., Huang J., Fu W., Xie X., Li W., Tang W., Xu C., Jin G. (2020). PCYT1A Suppresses Proliferation and Migration via Inhibiting mTORC1 Pathway in Lung Adenocarcinoma. Biochem. Biophys. Res. Commun..

[137] Xu X., Wang J., Xu L., Li P., Jiang P. (2024). P53 Suppresses Lipid Droplet-Fueled Tumorigenesis through Phosphatidylcholine. J. Clin. Investig..

[138] Lu F.C., Xie X.Y., Yin X.M., Gao Y.J. (2018). [Expression and potential clinical significance of cytidine triphosphate: Phosphocholine cytidylyltransferase-α in oral squamous cell carcinoma]. Zhonghua Kou Qiang Yi Xue Za Zhi/Chin. J. Stomatol..

[139] Xiong J., Wang L., Fei X.C., Jiang X.F., Zheng Z., Zhao Y., Wang C.F., Li B., Chen S.J., Janin A. (2017). MYC is a positive regulator of choline metabolism and impedes mitophagy-dependent necroptosis in diffuse large B-cell lymphoma. Blood Cancer J..

[140] Ross B.M., Moszczynska A., Peretti F.J., Adams V., Schmunk G.A., Kalasinsky K.S., Ang L., Mamalias N., Turenne S.D., Kish S.J. (2002). Decreased Activity of Brain Phospholipid Metabolic Enzymes in Human Users of Cocaine and Methamphetamine. Drug Alcohol Depend..

[141] Pati S., Ingram L.M., Sun M.K., Wagner J.J., Cummings B.S. (2019). Localization and Expression of CTP:Phosphocholine Cytidylyltransferase in Rat Brain Following Cocaine Exposure. J. Chem. Neuroanat..

[142] Renshaw P.F., Daniels S., Lundahl L.H., Rogers V., Lukas S.E. (1999). Short-Term Treatment with Citicoline (CDP-Choline) Attenuates Some Measures of Craving in Cocaine-Dependent Subjects: A Preliminary Report. Psychopharmacology.

[143] Ross B.M., Mamalias N., Moszczynska A., Rajput A.H., Kish S.J. (2001). Elevated Activity of Phospholipid Biosynthetic Enzymes in Substantia Nigra of Patients with Parkinson’s Disease. Neuroscience.

[144] Fernández-Irigoyen J., Cartas-Cejudo P., Iruarrizaga-Lejarreta M., Santamaría E. (2021). Alteration in the Cerebrospinal Fluid Lipidome in Parkinson’s Disease: A Post-Mortem Pilot Study. Biomedicines.

[145] O’Leary E.I., Jiang Z., Strub M.-P., Lee J.C. (2018). Effects of Phosphatidylcholine Membrane Fluidity on the Conformation and Aggregation of N-Terminally Acetylated α-Synuclein. J. Biol. Chem..

[146] Dou T., Kurouski D. (2022). Phosphatidylcholine and Phosphatidylserine Uniquely Modify the Secondary Structure of α-Synuclein Oligomers Formed in Their Presence at the Early Stages of Protein Aggregation. ACS Chem. Neurosci..

[147] Sarri E., Garcia-Dorado D., Abellan A., Soler-Soler J. (2006). Effects of Hypoxia, Glucose Deprivation and Acidosis on Phosphatidylcholine Synthesis in HL-1 Cardiomyocytes. CTP: Phosphocholine Cytidylyltransferase Activity Correlates with Sarcolemmal Disruption. Biochem. J..

[148] Montero-Bullon J.-F., Aveiro S.S., Melo T., Martins-Marques T., Lopes D., Neves B., Girão H., Domingues M.R.M., Domingues P. (2021). Cardiac Phospholipidome Is Altered during Ischemia and Reperfusion in an Ex Vivo Rat Model. Biochem. Biophys. Rep..

[149] Maliniak M.L., Cheriyan A.M., Sherman M.E., Liu Y., Gogineni K., Liu J., He J., Krishnamurti U., Miller-Kleinhenz J., Ashiqueali R. (2020). Detection of crown-like structures in breast adipose tissue and clinical outcomes among African-American and White women with breast cancer. Breast Cancer Res..

[150] Li X., Ren Y., Chang K., Wu W., Griffiths H.R., Lu S., Gao D. (2023). Adipose tissue macrophages as potential targets for obesity and metabolic diseases. Front. Immunol..

[151] Niebergall L.J., Jacobs R.L., Chaba T., Vance D.E. (2011). Phosphatidylcholine Protects against Steatosis in Mice but Not Non-Alcoholic Steatohepatitis. Biochim. Biophys. Acta.

[152] Li H., Zhu W., Zhang L., Lei H., Wu X., Guo L., Chen X., Wang Y., Tang H. (2015). The Metabolic Responses to Hepatitis B Virus Infection Shed New Light on Pathogenesis and Targets for Treatment. Sci. Rep..

[153] Huang Q., Lei H., Ding L., Wang Y. (2019). Stimulated Phospholipid Synthesis Is Key for Hepatitis B Virus Replications. Sci. Rep..

[154] Yu L., Zeng Z., Tan H., Feng Q., Zhou Q., Hu J., Li Y., Wang J., Yang W., Feng J. (2022). Significant Metabolic Alterations in Patients with Hepatitis B Virus Replication Observed via Serum Untargeted Metabolomics Shed New Light on Hepatitis B Virus Infection. J. Drug Target..

[155] Henneberry A.L., Wistow G., McMaster C.R. (2000). Cloning, Genomic Organization, and Characterization of a Human Cholinephosphotransferase. J. Biol. Chem..

[156] Butler P.L., Mallampalli R.K. (2010). Cross-Talk between Remodeling and De Novo Pathways Maintains Phospholipid Balance through Ubiquitination. J. Biol. Chem..

[157] Gong M., Liu X., Yang W., Song H., Zhao X., Ai X., Wang S., Wang H. (2021). Identification of a Lipid Metabolism-Associated Gene Signature Predicting Survival in Breast Cancer. Int. J. Gen. Med..

[158] Jia M., Andreassen T., Jensen L., Bathen T.F., Sinha I., Gao H., Zhao C., Haldosen L.-A., Cao Y., Girnita L. (2016). Estrogen Receptor α Promotes Breast Cancer by Reprogramming Choline Metabolism. Cancer Res..

[159] Chandrashekar D.S., Bashel B., Balasubramanya S.A.H., Creighton C.J., Ponce-Rodriguez I., Chakravarthi B.V.S.K., Varambally S. (2017). UALCAN: A Portal for Facilitating Tumor Subgroup Gene Expression and Survival Analyses. Neoplasia.

[160] Wen S., He Y., Wang L., Zhang J., Quan C., Niu Y., Huang H. (2020). Aberrant Activation of Super Enhancer and Choline Metabolism Drive Antiandrogen Therapy Resistance in Prostate Cancer. Oncogene.

[161] Chandrashekar D.S., Karthikeyan S.K., Korla P.K., Patel H., Shovon A.R., Athar M., Netto G.J., Qin Z.S., Kumar S., Manne U. (2022). UALCAN: An Update to the Integrated Cancer Data Analysis Platform. Neoplasia.

[162] Carrasco M.P., Segovia J.L., Marco C. (2001). Modulation of Biosynthesis of Phosphatidylcholine via CDP-Choline in Rat Liver: Influence of Ethanol on the Microsomal Cholinephosphotransferase Activity. Mol. Cell. Biochem..

[163] Fernando H., Bhopale K.K., Kondraganti S., Kaphalia B.S., Shakeel Ansari G.A. (2011). Lipidomic Changes in Rat Liver after Long-Term Exposure to Ethanol. Toxicol. Appl. Pharmacol..

[164] Tong R., Ding X., Liu F., Li H., Liu H., Song H., Wang Y., Zhang X., Liu S., Sun T. (2023). Classification of Subtypes and Identification of Dysregulated Genes in Sepsis. Front. Cell. Infect. Microbiol..

[165] Ahn W.-G., Jung J.-S., Kwon H.Y., Song D.-K. (2017). Alteration of Lysophosphatidylcholine-Related Metabolic Parameters in the Plasma of Mice with Experimental Sepsis. Inflammation.

[166] Tian Y., Pate C., Andreolotti A., Wang L., Tuomanen E., Boyd K., Claro E., Jackowski S. (2008). Cytokine Secretion Requires Phosphatidylcholine Synthesis. J. Cell Biol..

[167] Grove R.I., Allegretto N.J., Kiener P.A., Warr G.A. (1990). Lipopolysaccharide (LPS) Alters Phosphatidylcholine Metabolism in Elicited Peritoneal Macrophages. J. Leukoc. Biol..

[168] Bosch M.A., Risco C., Martin-Municio A., Bosch M.A., Risco C., Martin-Municio A. (1990). Effect of Escherichia Coli Lipopolysaccharide on Phosphatidylcholine Biosynthesis by Rat Lung and Alveolar Type II Cells. Mol. Cell. Biochem..

[169] Sabarirajan J., Vijayaraj P., Sarkar M., Nachiappan V. (2013). Effect of Lipopolysaccharide on Alteration of Phospholipids and Their Fatty Acid Composition in Spleen and Thymus by In Vitro Metabolic Labeling. J. Appl. Toxicol..

[170] Feng W., Tang R., Ye X., Xue C., Liao Y. (2016). Identification of Genes and Pathways Associated with Kidney Ischemia-Reperfusion Injury by Bioinformatics Analyses. Kidney Blood Press. Res..

[171] Rao S., Walters K.B., Wilson L., Chen B., Bolisetty S., Graves D., Barnes S., Agarwal A., Kabarowski J.H. (2016). Early Lipid Changes in Acute Kidney Injury Using SWATH Lipidomics Coupled with MALDI Tissue Imaging. Am. J. Physiol.-Ren. Physiol..

[172] Kenny T.C., Khan A., Son Y., Yue L., Heissel S., Sharma A., Pasolli H.A., Liu Y., Gamazon E.R., Alwaseem H. (2023). Integrative genetic analysis identifies FLVCR1 as a plasma-membrane choline transporter in mammals. Cell Metab..

[173] Ri K., Weng T.H., Claveras Cabezudo A., Jösting W., Zhang Y., Bazzone A., Leong N.C.P., Welsch S., Doty R.T., Gursu G. (2024). Molecular mechanism of choline and ethanolamine transport in humans. Nature.

[174] Son Y., Kenny T.C., Khan A., Birsoy K., Hite R.K. (2024). Structural basis of lipid head group entry to the Kennedy pathway by FLVCR1. Nature.

[175] Mercurio S., Aspesi A., Silengo L., Altruda F., Dianzani I., Chiabrando D. (2016). Alteration of heme metabolism in a cellular model of Diamond-Blackfan anemia. Eur. J. Haematol..

[176] Rajadhyaksha A.M., Elemento O., Puffenberger E.G., Schierberl K.C., Xiang J.Z., Putorti M.L., Berciano J., Poulin C., Brais B., Michaelides M. (2010). Mutations in FLVCR1 cause posterior column ataxia and retinitis pigmentosa. Am. J. Hum. Genet..

[177] Calame D.G., Wong J.H., Panda P., Nguyen D.T., Leong N.C.P., Sangermano R., Patankar S.G., Abdel-Hamid M.S., AlAbdi L., Safwat S. (2025). Biallelic variation in the choline and ethanolamine transporter FLVCR1 underlies a severe developmental disorder spectrum. Genet. Med..

[178] Rey M.A., Duffy S.P., Brown J.K., Kennedy J.A., Dick J.E., Dror Y., Tailor C.S. (2008). Enhanced alternative splicing of the FLVCR1 gene in Diamond Blackfan anemia disrupts FLVCR1 expression and function that are critical for erythropoiesis. Haematologica.

[179] Fiorito V., Neri F., Pala V., Silengo L., Oliviero S., Altruda F., Tolosano E. (2014). Hypoxia controls Flvcr1 gene expression in Caco2 cells through HIF2α and ETS1. Biochim. Biophys. Acta.

[180] Gao Q., Fan L., Chen Y., Cai J. (2022). Identification of the hub and prognostic genes in liver hepatocellular carcinoma via bioinformatics analysis. Front. Mol. Biosci..

[181] Peng C., Song Y., Chen W., Wang X., Liu X., Wang F., Wu D., Ma S., Wang X., Gao C. (2018). FLVCR1 promotes the proliferation and tumorigenicity of synovial sarcoma through inhibiting apoptosis and autophagy. Int. J. Oncol..

[182] Zhou S., Zhang M., Zhou C., Meng Y., Yang H., Ye W. (2021). FLVCR1 Predicts Poor Prognosis and Promotes Malignant Phenotype in Esophageal Squamous Cell Carcinoma via Upregulating CSE1L. Front. Oncol..

[183] Weinstein J.N., Collisson E.A., Mills G.B., Shaw K.R.M., Ozenberger B.A., Ellrott K., Shmulevich I., Sander C., Stuart J.M., Cancer Genome Atlas Research Network (2013). The Cancer Genome Atlas Pan-Cancer Analysis Project. Nat. Genet..

[184] Ridgway N.D., Vance D.E. (1988). Specificity of Rat Hepatic Phosphatidylethanolamine N-Methyltransferase for Molecular Species of Diacyl Phosphatidylethanolamine. J. Biol. Chem..

[185] Kuge O., Nishijima M., Akamatsu Y. (1991). A Cloned Gene Encoding Phosphatidylserine Decarboxylase Complements the Phosphatidylserine Biosynthetic Defect of a Chinese Hamster Ovary Cell Mutant. J. Biol. Chem..

